# Supplementary material for: A GntR family regulator HutC senses PCA to regulate histidine catabolism in Pseudomonas aeruginosa
Source: Microbiol Spectr. 2025 Nov 28;14(1):e00816-25. doi: 10.1128/spectrum.00816-25 (PMC12772324; doi:10.1128/spectrum.00816-25)
Supplement: Supplemental figures — Fig. S1 to S6. [file spectrum.00816-25-s0001.docx]

**HTH DNA binding domain**


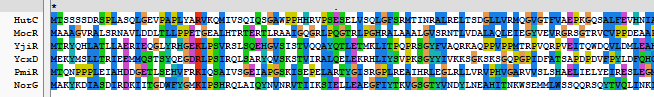


10 20 30 40 50 60 70 80 90 100

110 120 130 140 150 160 170 180 190 200 200


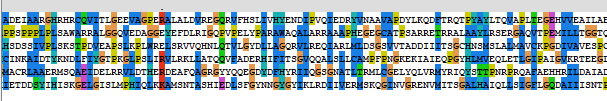

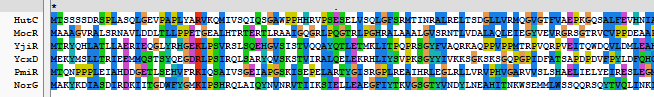

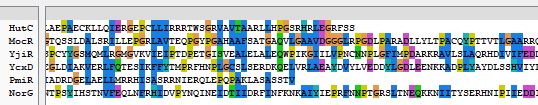


210 220 230 240 250 260 270 280 280

Fig.S1. Multiple alignments for HutC homologues. The protein sequences from different bacterial species are as follows: *Deinococcus radiodurans* (MocR), *Escherichia coli* (YjiR), *Bacillus subtilis* (YcxD), *Pseudomonas aeruginosa* (YjiR), *Staphylococcus aureus* (NorG). The putative conserved amino acid residues across the different proteins are indicated in the same colors. The Red box shows the putative DNA binding HTH domains.


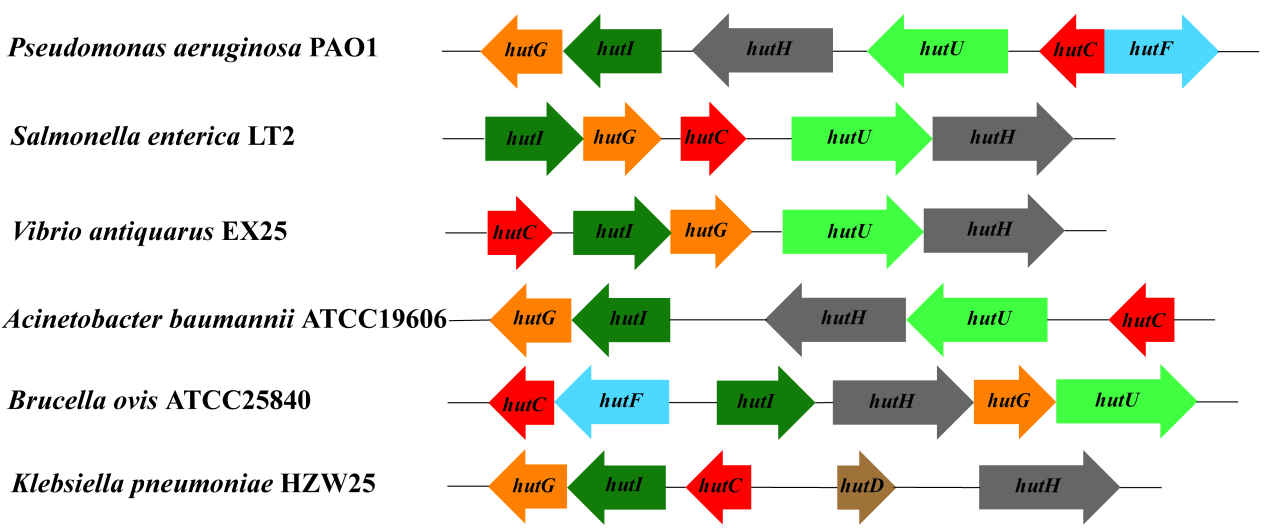


Fig. S2. Genetic organization of the histidine catabolism in *P. aeruginosa* PAO1 and five additional bacteria. The counterparts of the orthologues are shown in the same color and the direction of the arrow represents the direction of transcription. Their similarity to the sequence of *P. aeruginosa* PAO1 metabolic enzymes is listed.


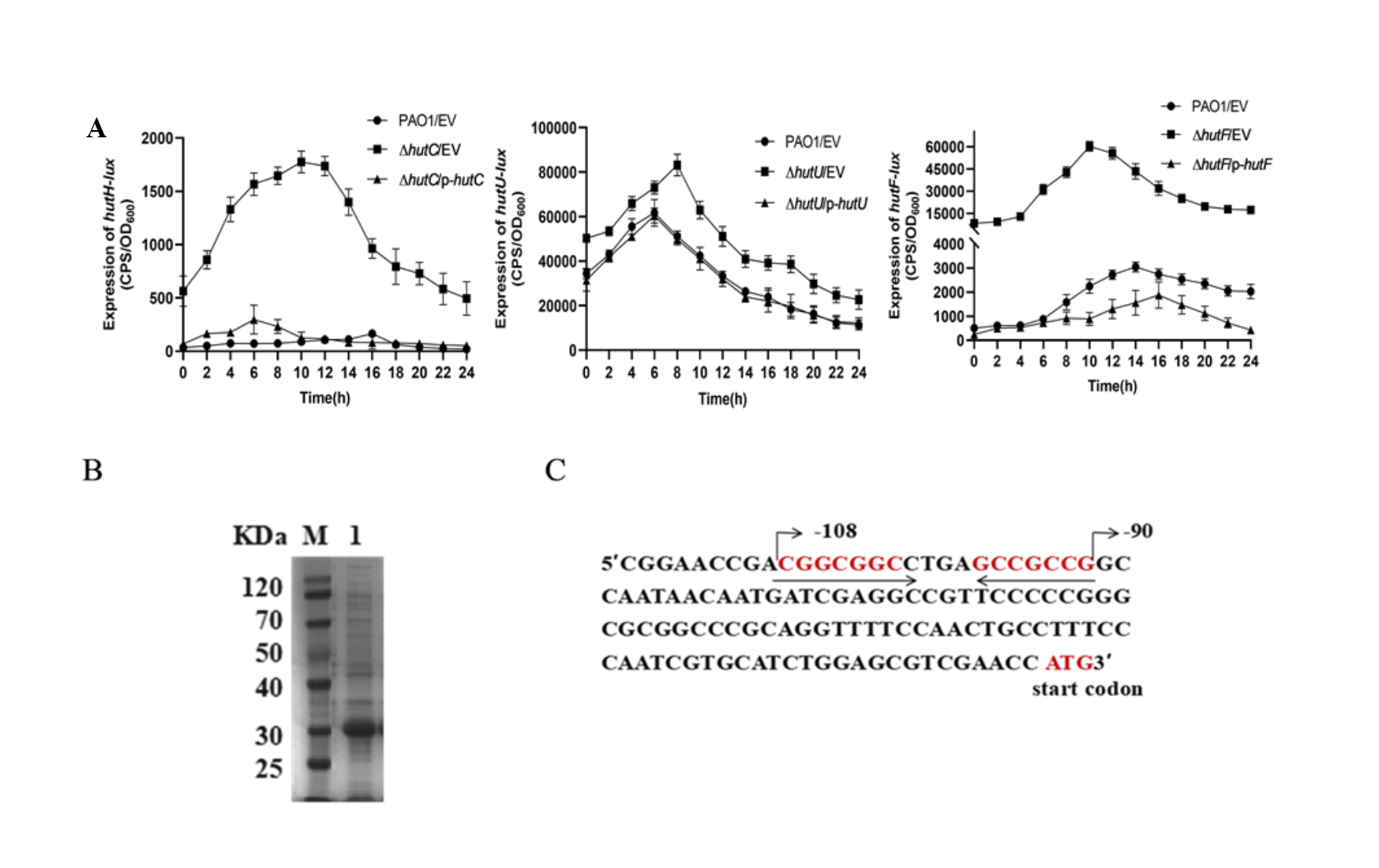


Fig. S3. (A) The promoter activity of *hutH*, *hutU* and *hutF* was measured in the wild-type PAO1, the Δ*hutC* mutant, and the complemented strain cultured in LB medium. Error bars indicate the SD of the mean from three independent experiments. (B) SDS-PAGE analysis of the expression and purification of HutC. Lane M, protein markers (25–120 kDa). Lane 1, purified recombinant HutC. (C) Sequence analysis of the promoter region upstream of the *hutG*, gene. The inverted repeat is represented in bold red font underlined by arrows. The start codon of *hutG* is shown in bold red font.


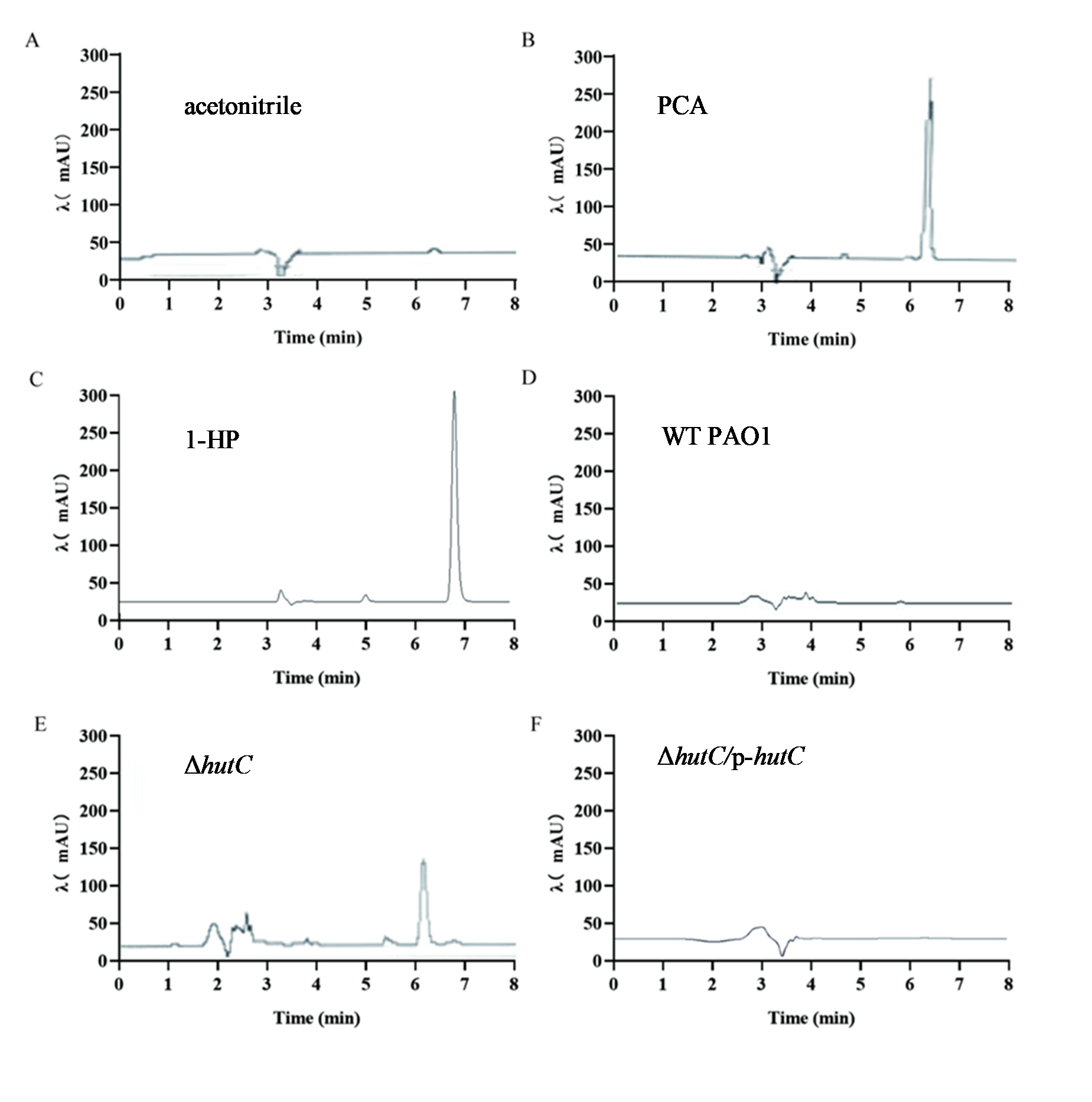


Fig. S4. HPLC chromatogram analysis of the standard PCA, 1-HP and the culture supernatant extract of WT PAO1, Δ*hutC* and its complemented strain (A) Acetonitrile. (B) Standard PCA. (C) Standard 1-HP. (D) The culture supernatant extract of WT PAO1. (E) The culture supernatant extract of Δ*hutC*. (F) The culture supernatant extract of Δ*hutC/*p-*hutC* .


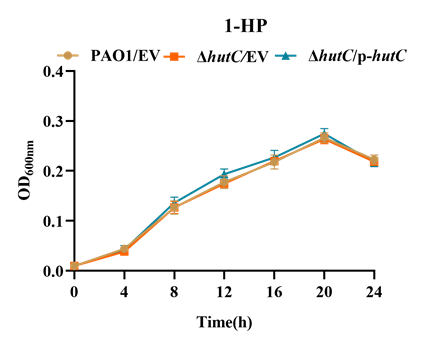


Fig. S5. The growth assay was conducted on the WT PAO1, Δ*hutC* and its complemented strains using minimal medium supplemented with 10 mM 1-HP as the sole carbon and nitrogen source. Complementation of the Δ*hutC* mutant by introducing the corresponding complemented plasmid restored wild-type phenotype. EV represents empty vector. Error bars indicate the SD of the mean from three independent experiments.


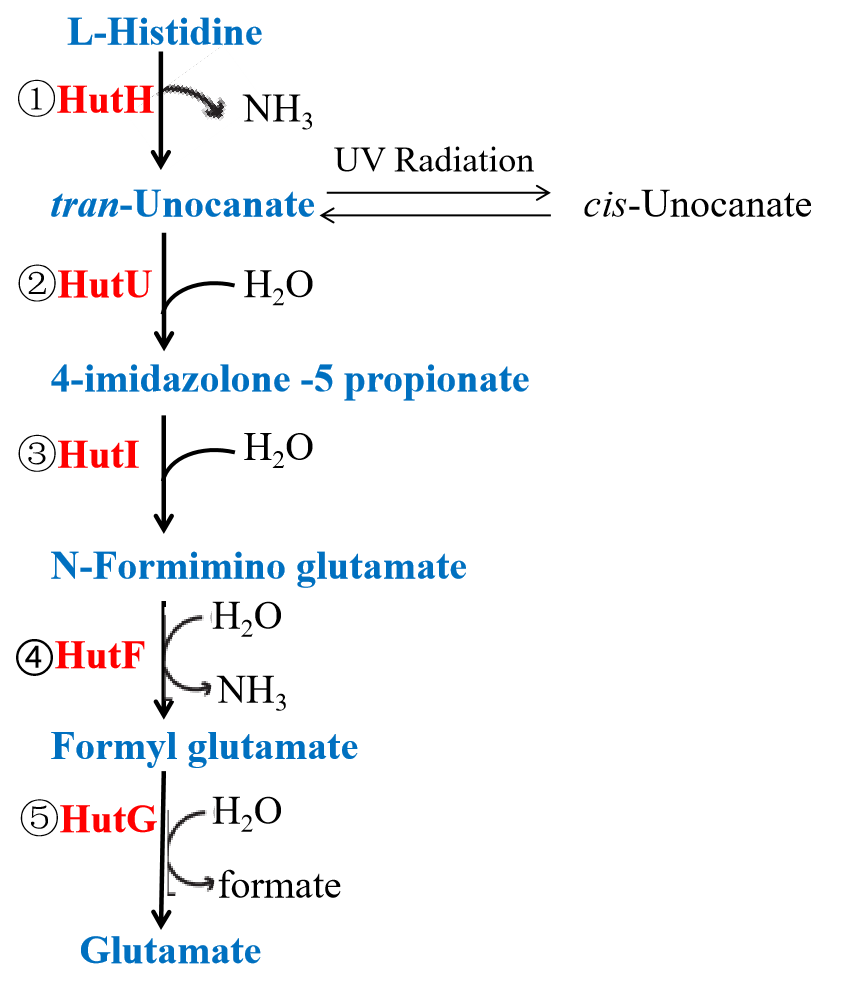


Fig. S6. Model of histidine catabolism in *P. aeruginosa*. Metabolites are represented by blue words. Enzymes are represented by red words
