# Supplementary material for: A GntR family regulator HutC senses PCA to regulate histidine catabolism in Pseudomonas aeruginosa
Source: Microbiol Spectr. 2025 Nov 28;14(1):e00816-25. doi: 10.1128/spectrum.00816-25 (PMC12772324; doi:10.1128/spectrum.00816-25)
Supplement: Supplemental tables — Tables S1 to S4. [file spectrum.00816-25-s0002.docx]

**Table S1. Genes with altered expression in the Δ*hutC* mutant and PAO1 (Δ*hutC* vs PAO1) by RNA-seq**

| Gene_ID | Gene name | log2. Fold change | *P*value | Function |
| --- | --- | --- | --- | --- |
| PA3993 | PA3993 | 8.216827 | 1.15E-10 | probable transposase |
| PA2111 | PA2111 | 6.434888 | 8.26E-34 | hypothetical protein |
| PA5098 | *hutH* | 6.167301 | 2.35E-80 | histidine ammonia-lyase |
| PA1210 | PA1210 | 5.503535 | 3.30E-16 | conserved hypothetical protein |
| PA3190 | PA3190 | 5.239487 | 6.85E-89 | probable binding protein component of ABC sugar transporter |
| PA5096 | PA5096 | 5.230445 | 5.36E-48 | probable binding protein component of ABC transporter |
| PA5097 | PA5097 | 5.165521 | 1.22E-49 | probable amino acid permease |
| PA2110 | PA2110 | 5.162239 | 9.22E-24 | hypothetical protein |
| PA2575 | PA2575 | 5.024901 | 3.05E-88 | hypothetical protein |
| PA2300 | *chiC* | 5.012215 | 3.41E-12 | chitinase |
| PA3240 | PA3240 | 4.991468 | 1.80E-47 | conserved hypothetical protein |
| PA2112 | PA2112 | 4.952852 | 2.85E-13 | conserved hypothetical protein |
| PA5093 | PA5093 | 4.722059 | 1.05E-58 | probable histidine/phenylalanine ammonia-lyase |
| PA5100 | *hutU* | 4.543380 | 9.04E-96 | urocanase |
| PA2116 | PA2116 | 4.471604 | 5.47E-34 | conserved hypothetical protein |
| PA2173 | PA2173 | 4.319092 | 1.62E-09 | hypothetical protein |
| PA4141 | PA4141 | 4.268573 | 3.17E-10 | hypothetical protein |
| PA1137 | PA1137 | 4.260069 | 9.61E-90 | probable oxidoreductase |
| PA5099 | PA5099 | 4.257189 | 4.22E-33 | probable transporter |
| PA4208 | *opmD* | 4.237004 | 2.20E-39 | probable outer membrane protein precursor |
| PA5092 | *hutI* | 4.225974 | 1.53E-60 | imidazolone-5-propionate hydrolase HutI |
| PA0132 | PA0132 | 4.186588 | 3.60E-41 | beta-alanine--pyruvate transaminase |
| PA2113 | *opdO* | 4.180647 | 3.46E-14 | pyroglutatmate porin OpdO |
| PA2864 | PA2864 | 4.148587 | 7.79E-08 | conserved hypothetical protein |
| PA3223 | *azoR3* | 4.132369 | 4.12E-31 | AzoR azoreductase 3 |
| PA0887 | *acsA* | 3.911927 | 1.45E-52 | acetyl-coenzyme A synthetase |
| PA5091 | *hutG* | 3.888480 | 9.83E-56 | N-formylglutamate amidohydrolase |
| PA2131 | *cupA4* | 3.817833 | 1.19E-12 | fimbrial subunit CupA4 |
| PA5106 | PA5106 | 3.723821 | 1.46E-93 | conserved hypothetical protein |
| PA3080 | PA3080 | 3.723361 | 3.69E-45 | hypothetical protein |
| PA5095 | PA5095 | 3.689003 | 1.59E-27 | probable permease of ABC transporter |
| PA4726 | *cbrB* | 3.675637 | 5.09E-74 | two-component response regulator CbrB |
| PA2069 | PA2069 | 3.674804 | 5.28E-34 | probable carbamoyl transferase |
| PA2132 | *cupA5* | 3.649040 | 9.73E-06 | chaperone CupA5 |
| PA2839 | PA2839 | 3.643512 | 1.68E-30 | conserved hypothetical protein |
| PA2552 | PA2552 | 3.631547 | 5.77E-49 | probable acyl-CoA dehydrogenase |
| PA3330 | PA3330 | 3.596537 | 2.41E-44 | probable short chain dehydrogenase |
| PA3569 | *mmsB* | 3.575724 | 6.73E-21 | 3-hydroxyisobutyrate dehydrogenase |
| PA5104 | PA5104 | 3.528863 | 1.44E-72 | conserved hypothetical protein |
| PA4134 | PA4134 | 3.495724 | 7.33E-42 | hypothetical protein |
| PA2610 | PA2610 | 3.490136 | 1.07E-31 | conserved hypothetical protein |
| PA1869 | PA1869 | 3.479633 | 1.67E-41 | probable acyl carrier protein |
| PA2555 | PA2555 | 3.453965 | 9.43E-58 | probable AMP-binding enzyme |
| PA3329 | PA3329 | 3.439026 | 2.18E-32 | hypothetical protein |
| PA2360 | PA2360 | 3.345028 | 3.39E-30 | hypothetical protein |
| PA0131 | PA0131 | 3.341627 | 6.84E-11 | hypothetical protein |
| PA3334 | PA3334 | 3.333905 | 5.16E-09 | probable acyl carrier protein |
| PA2114 | PA2114 | 3.323617 | 1.41E-27 | probable major facilitator superfamily (MFS) transporter |
| PA5094 | PA5094 | 3.321448 | 5.46E-31 | probable ATP-binding component of ABC transporter |
| PA4525 | *pilA* | 3.319207 | 6.87E-36 | type 4 fimbrial precursor PilA |
| PA3187 | PA3187 | 3.295860 | 5.98E-20 | probable ATP-binding component of ABC transporter |
| PA2553 | PA2553 | 3.276390 | 7.11E-35 | probable acyl-CoA thiolase |
| PA4757 | PA4757 | 3.271155 | 2.93E-46 | conserved hypothetical protein |
| PA3333 | *fabH2* | 3.264880 | 2.22E-22 | 3-oxoacyl-[acyl-carrier-protein] synthase III |
| PA2109 | PA2109 | 3.254113 | 5.53E-05 | hypothetical protein |
| PA0872 | *phhA* | 3.248268 | 8.87E-59 | phenylalanine-4-hydroxylase |
| PA3478 | *rhlB* | 3.248122 | 1.75E-20 | rhamnosyltransferase chain B |
| PA3328 | PA3328 | 3.239862 | 2.97E-31 | probable FAD-dependent monooxygenase |
| PA4683 | PA4683 | 3.228367 | 6.22E-37 | hypothetical protein |
| PA2646 | *nuoK* | 3.227456 | 3.02E-27 | NADH dehydrogenase I chain K |
| PA1244 | PA1244 | 3.189270 | 3.16E-56 | hypothetical protein |
| PA2554 | PA2554 | 3.184288 | 1.40E-38 | probable short-chain dehydrogenase |
| PA5040 | *pilQ* | 3.178282 | 5.58E-62 | Type 4 fimbrial biogenesis outer membrane protein PilQ precursor |
| PA2649 | *nuoN* | 3.148223 | 3.46E-47 | NADH dehydrogenase I chain N |
| PA2068 | PA2068 | 3.128217 | 6.46E-10 | probable major facilitator superfamily (MFS) transporter |
| PA1000 | *pqsE* | 3.104239 | 1.57E-33 | Quinolone signal response protein |
| PA0678 | PA0678 | 3.087393 | 0.00240 | HxcU putative pseudopilin |
| PA2274 | PA2274 | 3.080768 | 2.24E-10 | hypothetical protein |
| PA2052 | *cynS* | 3.057187 | 6.16E-16 | cyanate lyase |
| PA2098 | PA2098 | 3.044033 | 0.00814 | probable esterase/deacetylase |
| PA1871 | *lasA* | 3.039838 | 1.33E-32 | LasA protease precursor |
| PA2067 | PA2067 | 3.035670 | 4.85E-09 | probable hydrolase |
| PA4206 | *mexH* | 3.035190 | 3.76E-24 | probable Resistance-Nodulation-Cell Division (RND) efflux membrane fusion protein precursor |
| PA4758 | *carA* | 3.003553 | 1.48E-56 | carbamoyl-phosphate synthase small chain |
| PA3332 | PA3332 | 2.994176 | 1.38E-17 | conserved hypothetical protein |
| PA2029 | PA2029 | 2.987644 | 0.00023 | hypothetical protein |
| PA2647 | *nuoL* | 2.985396 | 2.67E-39 | NADH dehydrogenase I chain L |
| PA4133 | PA4133 | 2.973489 | 2.66E-29 | cytochrome c oxidase subunit (cbb3-type) |
| PA1887 | PA1887 | 2.972312 | 4.45E-36 | hypothetical protein |
| PA4889 | PA4889 | 2.954509 | 6.03E-20 | probable oxidoreductase |
| PA2442 | *gcvT2* | 2.951229 | 2.11E-37 | glycine cleavage system protein T2 |
| PA4132 | PA4132 | 2.947117 | 3.89E-39 | conserved hypothetical protein |
| PA0952 | PA0952 | 2.930673 | 9.96E-26 | hypothetical protein |
| PA1548 | PA1548 | 2.917437 | 3.31E-22 | conserved hypothetical protein |
| PA0951 | PA0951 | 2.898533 | 1.23E-29 | probable ribonuclease |
| PA2408 | PA2408 | 2.898467 | 0.00380 | probable ATP-binding component of ABC transporter |
| PA3009 | PA3009 | 2.827473 | 2.69E-19 | hypothetical protein |
| PA2642 | *nuoG* | 2.823706 | 1.08E-50 | NADH dehydrogenase I chain G |
| PA2092 | PA2092 | 2.802222 | 3.13E-07 | probable major facilitator superfamily (MFS) transporter |
| PA1584 | *sdhB* | 2.800607 | 7.52E-40 | succinate dehydrogenase (B subunit) |
| PA2648 | *nuoM* | 2.798310 | 2.60E-43 | NADH dehydrogenase I chain M |
| PA2195 | *hcnC* | 2.796799 | 5.59E-37 | hydrogen cyanide synthase HcnC |
| PA1478 | PA1478 | 2.783379 | 0.05943 | hypothetical protein |
| PA3808 | PA3808 | 2.778933 | 9.46E-21 | conserved hypothetical protein |
| PA3479 | *rhlA* | 2.776355 | 9.03E-18 | rhamnosyltransferase chain A |
| PA2567 | PA2567 | 2.776037 | 1.34E-32 | hypothetical protein |
| PA4888 | *desB* | 2.762630 | 8.89E-15 | acyl-CoA delta-9-desaturase%2C DesB |
| PA4756 | *carB* | 2.746136 | 4.89E-42 | carbamoylphosphate synthetase large subunit |
| PA0865 | *hpd* | 2.744537 | 1.60E-28 | 4-hydroxyphenylpyruvate dioxygenase |
| PA3494 | PA3494 | 2.743716 | 8.23E-09 | conserved hypothetical protein |
| PA2444 | *glyA2* | 2.740836 | 4.93E-21 | serine hydroxymethyltransferase |
| PA0526 | PA0526 | 2.739569 | 0.02520 | hypothetical protein |
| PA3971 | PA3971 | 2.695412 | 3.25E-13 | hypothetical protein |
| PA2674 | PA2674 | 2.694281 | 0.06882 | probable type II secretion system protein |
| PA3073 | PA3073 | 2.684126 | 2.18E-20 | hypothetical protein |
| PA2162 | PA2162 | 2.682327 | 2.58E-15 | probable glycosyl hydrolase |
| PA0793 | PA0793 | 2.662693 | 2.01E-20 | hypothetical protein |
| PA2163 | PA2163 | 2.637270 | 1.48E-13 | hypothetical protein |
| PA3075 | PA3075 | 2.632441 | 1.71E-26 | hypothetical protein |
| PA3327 | PA3327 | 2.631660 | 1.54E-36 | probable non-ribosomal peptide synthetase |
| PA3099 | *xcpV* | 2.626722 | 0.00042 | general secretion pathway protein I |
| PA1711 | *exsE* | 2.622102 | 4.30E-06 | ExsE |
| PA5367 | *pstA* | 2.617560 | 3.93E-31 | membrane protein component of ABC phosphate transporter |
| PA3485 | PA3485 | 2.617434 | 3.84E-06 | hypothetical protein |
| PA3266 | *capB* | 2.616937 | 2.02E-26 | cold acclimation protein B |
| PA1274 | PA1274 | 2.615720 | 0.00017 | conserved hypothetical protein |
| PA3493 | PA3493 | 2.614746 | 1.39E-10 | conserved hypothetical protein |
| PA4755 | *greA* | 2.611558 | 1.13E-40 | transcription elongation factor GreA |
| PA0852 | *cbpD* | 2.608965 | 8.91E-20 | chitin-binding protein CbpD precursor |
| PA5220 | PA5220 | 2.600797 | 1.21E-23 | hypothetical protein |
| PA4128 | PA4128 | 2.600408 | 5.96E-05 | conserved hypothetical protein |
| PA2049 | PA2049 | 2.597590 | 1.55E-16 | hypothetical protein |
| PA4528 | *pilD* | 2.589301 | 5.01E-27 | type 4 prepilin peptidase PilD |
| PA4357 | PA4357 | 2.585127 | 1.61E-19 | conserved hypothetical protein |
| PA2643 | *nuoH* | 2.584879 | 2.60E-46 | NADH dehydrogenase I chain H |
| PA2327 | PA2327 | 2.582917 | 3.35E-36 | probable permease of ABC transporter |
| PA2641 | *nuoF* | 2.576600 | 1.68E-35 | NADH dehydrogenase I chain F |
| PA5042 | *pilO* | 2.572008 | 5.60E-32 | type 4 fimbrial biogenesis protein PilO |
| PA0794 | PA0794 | 2.571347 | 4.36E-24 | probable aconitate hydratase |
| PA0534 | PA0534 | 2.545523 | 1.35E-34 | conserved hypothetical protein |
| PA3860 | PA3860 | 2.538235 | 1.36E-19 | probable AMP-binding enzyme |
| PA2445 | *gcvP2* | 2.536871 | 4.82E-36 | glycine cleavage system protein P2 |
| PA4659 | PA4659 | 2.531246 | 1.41E-11 | probable transcriptional regulator |
| PA0713 | PA0713 | 2.525452 | 2.99E-14 | hypothetical protein |
| PA4844 | PA4844 | 2.518412 | 5.66E-22 | probable chemotaxis transducer |
| PA3972 | PA3972 | 2.504215 | 5.32E-26 | probable acyl-CoA dehydrogenase |
| PA2566 | PA2566 | 2.487532 | 3.87E-22 | conserved hypothetical protein |
| PA3922 | PA3922 | 2.481268 | 9.95E-05 | conserved hypothetical protein |
| PA3331 | PA3331 | 2.473498 | 1.83E-23 | cytochrome P450 |
| PA3933 | PA3933 | 2.454597 | 1.82E-29 | probable choline transporter |
| PA1656 | PA1656 | 2.451071 | 6.12E-26 | hypothetical protein |
| PA2679 | PA2679 | 2.446556 | 3.02E-24 | hypothetical protein |
| PA1248 | *aprF* | 2.445397 | 4.55E-14 | Alkaline protease secretion outer membrane protein AprF precursor |
| PA4207 | *mexI* | 2.439624 | 9.20E-24 | probable Resistance-Nodulation-Cell Division (RND) efflux transporter |
| PA3163 | *cmk* | 2.418673 | 2.58E-25 | cytidylate kinase |
| PA2066 | PA2066 | 2.415706 | 2.35E-07 | hypothetical protein |
| PA2194 | *hcnB* | 2.414843 | 3.99E-16 | hydrogen cyanide synthase HcnB |
| PA0413 | *chpA* | 2.414769 | 5.31E-55 | component of chemotactic signal transduction system |
| PA2644 | *nuoI* | 2.413335 | 3.83E-18 | NADH Dehydrogenase I chain I |
| PA1707 | *pcrH* | 2.412420 | 3.98E-08 | regulatory protein PcrH |
| PA3271 | PA3271 | 2.406487 | 1.35E-30 | probable two-component sensor |
| PA2074 | PA2074 | 2.401320 | 0.01272 | hypothetical protein |
| PA2436 | PA2436 | 2.397613 | 7.32E-11 | hypothetical protein |
| PA5090 | PA5090 | 2.397230 | 7.35E-18 | conserved hypothetical protein |
| PA2161 | PA2161 | 2.394742 | 0.02756 | hypothetical protein |
| PA2381 | PA2381 | 2.392967 | 3.47E-25 | hypothetical protein |
| PA0523 | *norC* | 2.386862 | 0.12579 | nitric-oxide reductase subunit C |
| PA0130 | PA0130 | 2.383411 | 2.15E-14 | probable aldehyde dehydrogenase |
| PA2044 | PA2044 | 2.383031 | 2.32E-13 | hypothetical protein |
| PA2250 | *lpdV* | 2.382628 | 9.00E-21 | lipoamide dehydrogenase-Val |
| PA3621 | *fdxA* | 2.380303 | 4.18E-37 | ferredoxin I |
| PA0414 | *chpB* | 2.379101 | 2.54E-26 | probable methylesterase |
| PA3920 | PA3920 | 2.378744 | 1.56E-12 | probable metal transporting P-type ATPase |
| PA3570 | *mmsA* | 2.377318 | 1.67E-12 | methylmalonate-semialdehyde dehydrogenase |
| PA4139 | PA4139 | 2.376181 | 0.00010 | hypothetical protein |
| PA5543 | PA5543 | 2.373168 | 0.25509 | hypothetical protein |
| PA3394 | *nosF* | 2.365791 | 0.05211 | NosF protein |
| PA3074 | PA3074 | 2.360055 | 3.25E-21 | hypothetical protein |
| PA0714 | PA0714 | 2.357093 | 8.55E-16 | hypothetical protein |
| PA2788 | PA2788 | 2.356258 | 1.34E-21 | probable chemotaxis transducer |
| PA2141 | PA2141 | 2.356213 | 0.01554 | hypothetical protein |
| PA1847 | PA1847 | 2.354536 | 3.19E-14 | conserved hypothetical protein |
| PA4660 | *phr* | 2.350754 | 0.00114 | deoxyribodipyrimidine photolyase |
| PA0792 | *prpD* | 2.348943 | 1.00E-18 | propionate catabolic protein PrpD |
| PA2280 | PA2280 | 2.344872 | 5.82E-09 | conserved hypothetical protein |
| PA3392 | *nosZ* | 2.337311 | 0.06302 | nitrous-oxide reductase precursor |
| PA3072 | PA3072 | 2.334379 | 8.70E-05 | hypothetical protein |
| PA2645 | *nuoJ* | 2.326818 | 3.48E-13 | NADH dehydrogenase I chain J |
| PA1247 | *aprE* | 2.321252 | 1.71E-11 | alkaline protease secretion protein AprE |
| PA4129 | PA4129 | 2.314905 | 7.00E-23 | hypothetical protein |
| PA4601 | morA | 2.313364 | 3.87E-24 | motility regulator |
| PA2407 | PA2407 | 2.312469 | 0.00444 | probable adhesion protein |
| PA2088 | PA2088 | 2.310956 | 6.15E-07 | hypothetical protein |
| PA5369 | *pstS* | 2.303606 | 5.72E-16 | phosphate ABC transporter periplasmic phosphate-binding protein PstS |
| PA3396 | *nosL* | 2.302559 | 0.00194 | NosL protein |
| PA4530 | PA4530 | 2.301760 | 5.13E-12 | conserved hypothetical protein |
| PA3195 | *gapA* | 2.291679 | 1.01E-25 | glyceraldehyde 3-phosphate dehydrogenase |
| PA1550 | PA1550 | 2.289992 | 3.89E-37 | hypothetical protein |
| PA1183 | *dctA* | 2.289733 | 1.86E-31 | C4-dicarboxylate transport protein |
| PA2236 | *pslF* | 2.286473 | 6.61E-21 | PslF |
| PA2172 | PA2172 | 2.281490 | 1.92E-09 | hypothetical protein |
| PA3591 | PA3591 | 2.281035 | 0.07004 | probable enoyl-CoA hydratase/isomerase |
| PA2328 | PA2328 | 2.279577 | 1.42E-16 | hypothetical protein |
| PA2130 | *cupA3* | 2.276637 | 1.37E-11 | usher CupA3 |
| PA3393 | *nosD* | 2.270625 | 0.05180 | NosD protein |
| PA0021 | PA0021 | 2.259667 | 9.44E-06 | conserved hypothetical protein |
| PA1624 | PA1624 | 2.258735 | 6.04E-18 | hypothetical protein |
| PA1583 | *sdhA* | 2.257887 | 3.31E-30 | succinate dehydrogenase (A subunit) |
| PA0524 | *norB* | 2.257614 | 0.09344 | nitric-oxide reductase subunit B |
| PA4182 | PA4182 | 2.255029 | 3.97E-15 | hypothetical protein |
| PA2144 | *glgP* | 2.254955 | 2.50E-13 | glycogen phosphorylase |
| PA1130 | *rhlC* | 2.251915 | 1.12E-19 | rhamnosyltransferase 2 |
| PA3789 | PA3789 | 2.251662 | 1.80E-22 | hypothetical protein |
| PA2238 | *pslH* | 2.249531 | 1.08E-20 | PslH |
| PA2169 | PA2169 | 2.240341 | 1.21E-08 | hypothetical protein |
| PA1549 | PA1549 | 2.232742 | 1.55E-29 | probable cation-transporting P-type ATPase |
| PA1585 | *sucA* | 2.223550 | 2.28E-33 | 2-oxoglutarate dehydrogenase (E1 subunit) |
| PA2342 | *mtlD* | 2.217610 | 0.00310 | mannitol dehydrogenase |
| PA3233 | PA3233 | 2.212782 | 4.45E-12 | hypothetical protein |
| PA1335 | PA1335 | 2.212208 | 3.90E-11 | probable two-component response regulator |
| PA1587 | *lpdG* | 2.205817 | 6.95E-34 | lipoamide dehydrogenase-glc |
| PA0050 | PA0050 | 2.199473 | 0.00393 | hypothetical protein |
| PA5321 | *dut* | 2.199355 | 8.83E-25 | deoxyuridine 5'-triphosphate nucleotidohydrolase |
| PA0870 | *phhC* | 2.198471 | 1.61E-27 | aromatic amino acid aminotransferase |
| PA1586 | *sucB* | 2.190684 | 6.64E-39 | dihydrolipoamide succinyltransferase (E2 subunit) |
| PA2437 | PA2437 | 2.189568 | 0.00013 | hypothetical protein |
| PA2640 | *nuoE* | 2.186438 | 3.03E-28 | NADH dehydrogenase I chain E |
| PA2180 | PA2180 | 2.173589 | 4.79E-09 | hypothetical protein |
| PA3241 | PA3241 | 2.173485 | 6.33E-11 | hypothetical protein |
| PA2155 | PA2155 | 2.171867 | 2.23E-05 | probable phospholipase |
| PA4658 | PA4658 | 2.169436 | 0.00043 | hypothetical protein |
| PA5368 | *pstC* | 2.164877 | 1.10E-20 | membrane protein component of ABC phosphate transporter |
| PA2171 | PA2171 | 2.163065 | 6.05E-13 | hypothetical protein |
| PA3424 | PA3424 | 2.159445 | 6.47E-14 | hypothetical protein |
| PA2391 | *opmQ* | 2.151199 | 0.00660 | probable outer membrane protein precursor |
| PA1135 | PA1135 | 2.148843 | 1.80E-14 | conserved hypothetical protein |
| PA0927 | *ldhA* | 2.143741 | 3.58E-09 | D-lactate dehydrogenase (fermentative) |
| PA3425 | PA3425 | 2.142475 | 3.09E-07 | hypothetical protein |
| PA4552 | *pilW* | 2.140230 | 7.59E-10 | type 4 fimbrial biogenesis protein PilW |
| PA4324 | PA4324 | 2.139972 | 6.43E-25 | hypothetical protein |
| PA2031 | PA2031 | 2.131557 | 0.00014 | hypothetical protein |
| PA0017 | PA0017 | 2.130714 | 3.30E-18 | conserved hypothetical protein |
| PA1853 | PA1853 | 2.130382 | 1.27E-15 | probable transcriptional regulator |
| PA3091 | PA3091 | 2.121361 | 1.62E-19 | hypothetical protein |
| PA1131 | PA1131 | 2.121334 | 1.10E-16 | probable major facilitator superfamily (MFS) transporter |
| PA4615 | PA4615 | 2.117689 | 4.19E-11 | probable oxidoreductase |
| PA1888 | PA1888 | 2.116809 | 3.99E-17 | hypothetical protein |
| PA5041 | *pilP* | 2.116169 | 8.30E-16 | type 4 fimbrial biogenesis protein PilP |
| PA2233 | *pslC* | 2.116058 | 4.83E-17 | PslC |
| PA2095 | PA2095 | 2.112617 | 0.01722 | hypothetical protein |
| PA4215 | *phzF1* | 2.111715 | 4.89E-09 | probable phenazine biosynthesis protein |
| PA1904 | *phzF2* | 2.111715 | 4.89E-09 | probable phenazine biosynthesis protein |
| PA1589 | *sucD* | 2.111084 | 2.52E-29 | succinyl-CoA synthetase alpha chain |
| PA0802 | PA0802 | 2.109004 | 0.21036 | hypothetical protein |
| PA3049 | *rmf* | 2.108709 | 1.18E-15 | ribosome modulation factor |
| PA3784 | PA3784 | 2.106137 | 9.33E-17 | hypothetical protein |
| PA3973 | PA3973 | 2.086579 | 7.81E-20 | probable transcriptional regulator |
| PA1279 | *cobU* | 2.084461 | 2.32E-07 | nicotinate-nucleotide--dimethylbenzimidazole phosphoribosyltransferase |
| PA5065 | *ubiB* | 2.080190 | 1.32E-15 | ubiquinone biosynthetic protein UbiB |
| PA2043 | PA2043 | 2.079536 | 1.34E-08 | hypothetical protein |
| PA0391 | PA0391 | 2.071579 | 1.06E-16 | hypothetical protein |
| PA0412 | *pilK* | 2.069111 | 1.99E-16 | methyltransferase PilK |
| PA1705 | *pcrG* | 2.066815 | 6.63E-07 | regulator in type III secretion |
| PA3361 | *lecB* | 2.065746 | 9.39E-11 | fucose-binding lectin PA-IIL |
| PA2128 | *cupA1* | 2.060348 | 6.79E-26 | fimbrial subunit CupA1 |
| PA2609 | PA2609 | 2.059312 | 1.00E-19 | hypothetical protein |
| PA5365 | *phoU* | 2.058971 | 1.86E-18 | phosphate uptake regulatory protein PhoU |
| PA2371 | PA2371 | 2.058477 | 5.53E-18 | probable ClpA/B-type protease |
| PA2239 | *pslI* | 2.057533 | 2.74E-18 | PslI |
| PA1059 | PA1059 | 2.054560 | 1.84E-09 | conserved hypothetical protein |
| PA2370 | PA2370 | 2.053401 | 7.54E-05 | hypothetical protein |
| PA3431 | PA3431 | 2.053316 | 7.35E-13 | conserved hypothetical protein |
| PA0745 | PA0745 | 2.051349 | 7.39E-15 | probable enoyl-CoA hydratase/isomerase |
| PA4523 | PA4523 | 2.048769 | 2.79E-25 | hypothetical protein |
| PA5288 | *glnK* | 2.047729 | 8.98E-20 | nitrogen regulatory protein P-II 2 |
| PA2471 | PA2471 | 2.044721 | 1.39E-05 | conserved hypothetical protein |
| PA3688 | PA3688 | 2.041800 | 1.09E-14 | hypothetical protein |
| PA3391 | *nosR* | 2.039490 | 0.11710 | regulatory protein NosR |
| PA2763 | PA2763 | 2.037087 | 6.74E-05 | hypothetical protein |
| PA0796 | *prpB* | 2.036527 | 5.15E-15 | carboxyphosphonoenolpyruvate phosphonomutase |
| PA5043 | *pilN* | 2.032157 | 7.24E-25 | type 4 fimbrial biogenesis protein PilN |
| PA5139 | PA5139 | 2.027979 | 1.39E-10 | hypothetical protein |
| PA0446 | PA0446 | 2.027558 | 1.29E-07 | conserved hypothetical protein |
| PA1708 | popB | 2.017891 | 1.46E-09 | translocator protein PopB |
| PA2143 | PA2143 | 2.011129 | 1.09E-07 | hypothetical protein |
| PA2237 | *pslG* | 2.010091 | 1.34E-17 | PslG |
| PA1337 | *ansB* | 2.009962 | 3.79E-15 | glutaminase-asparaginase |
| PA2036 | PA2036 | 2.000950 | 0.02273 | hypothetical protein |
| PA0574 | PA0574 | -2.000096 | 1.77E-17 | hypothetical protein |
| PA0884 | PA0884 | -2.005129 | 1.57E-09 | probable C4-dicarboxylate-binding periplasmic protein |
| PA0824 | PA0824 | -2.006060 | 0.00029 | hypothetical protein |
| PA0453 | PA0453 | -2.006998 | 1.10E-06 | hypothetical protein |
| PA5219 | PA5219 | -2.017055 | 4.61E-22 | hypothetical protein |
| PA3152 | *hisH2* | -2.023263 | 2.00E-10 | glutamine amidotransferase |
| PA2715 | PA2715 | -2.024844 | 0.18872 | probable ferredoxin |
| PA1635 | *kdpC* | -2.028448 | 0.01698 | potassium-transporting ATPase C chain |
| PA3284 | PA3284 | -2.030803 | 3.10E-08 | hypothetical protein |
| PA0069 | PA0069 | -2.033817 | 3.77E-14 | conserved hypothetical protein |
| PA4581 | *rtcR* | -2.034939 | 1.16E-17 | transcriptional regulator RtcR |
| PA1516 | PA1516 | -2.038846 | 3.00E-08 | hypothetical protein |
| PA1634 | *kdpB* | -2.040558 | 7.57E-09 | potassium-transporting ATPase B chain |
| PA2275 | PA2275 | -2.040978 | 5.37E-11 | probable alcohol dehydrogenase (Zn-dependent) |
| PA0817 | PA0817 | -2.042027 | 0.00025 | probable ring-cleaving dioxygenase |
| PA2855 | PA2855 | -2.044417 | 1.46E-10 | hypothetical protein |
| PA4741 | *rpsO* | -2.046355 | 3.89E-22 | 30S ribosomal protein S15 |
| PA2938 | PA2938 | -2.052450 | 3.48E-17 | probable transporter |
| PA0923 | *dinB* | -2.053184 | 5.66E-30 | DNA Polymerase IV DinB |
| PA0241 | PA0241 | -2.055387 | 0.00328 | probable major facilitator superfamily (MFS) transporter |
| PA2513 | *antB* | -2.056112 | 0.00950 | anthranilate dioxygenase small subunit |
| PA3741 | PA3741 | -2.057412 | 6.78E-09 | hypothetical protein |
| PA1150 | *pys2* | -2.059433 | 5.00E-21 | pyocin S2 |
| PA3262 | PA3262 | -2.060871 | 7.73E-29 | probable peptidyl-prolyl cis-trans isomerase FkbP-type |
| PA5327 | PA5327 | -2.062129 | 1.13E-07 | probable oxidoreductase |
| PA1030 | PA1030 | -2.065012 | 5.10E-19 | hypothetical protein |
| PA0119 | PA0119 | -2.066788 | 1.44E-12 | probable dicarboxylate transporter |
| PA0014 | PA0014 | -2.067470 | 1.12E-11 | hypothetical protein |
| PA1285 | PA1285 | -2.079130 | 7.93E-13 | probable transcriptional regulator |
| PA3553 | *arnC* | -2.087204 | 1.42E-10 | ArnC |
| PA1979 | *eraS* | -2.087325 | 0.46340 | sensor kinase EraS |
| PA0692 | PA0692 | -2.088299 | 6.76E-07 | hypothetical protein |
| PA1230 | PA1230 | -2.088978 | 2.60E-09 | hypothetical protein |
| PA0155 | *pcaR* | -2.096798 | 7.40E-07 | transcriptional regulator PcaR |
| PA1098 | *fleS* | -2.101381 | 1.03E-29 | two-component sensor |
| PA0201 | PA0201 | -2.101408 | 2.79E-16 | hypothetical protein |
| PA3414 | PA3414 | -2.104601 | 4.87E-16 | hypothetical protein |
| PA0624 | PA0624 | -2.104960 | 1.16E-08 | hypothetical protein |
| PA3732 | PA3732 | -2.107537 | 2.71E-22 | conserved hypothetical protein |
| PA3554 | *arnA* | -2.107676 | 1.80E-16 | ArnA |
| PA3731 | PA3731 | -2.111411 | 5.41E-26 | conserved hypothetical protein |
| PA3672 | PA3672 | -2.115310 | 1.50E-15 | probable ATP-binding component of ABC transporter |
| PA2886 | *atuA* | -2.123471 | 1.07E-13 | expressed protein with apparent function in citronellol catabolism |
| PA0167 | PA0167 | -2.128965 | 1.52E-12 | probable transcriptional regulator |
| PA0376 | *rpoH* | -2.130077 | 9.10E-38 | sigma factor RpoH |
| PA5566 | PA5566 | -2.132435 | 3.76E-05 | hypothetical protein |
| PA0845 | PA0845 | -2.134795 | 1.16E-13 | conserved hypothetical protein |
| PA1503 | PA1503 | -2.137327 | 5.73E-06 | hypothetical protein |
| PA2767 | PA2767 | -2.145445 | 9.99E-07 | probable enoyl-CoA hydratase/isomerase |
| PA5375 | *betT1* | -2.148709 | 2.30E-20 | choline transporter BetT |
| PA4563 | *rpsT* | -2.148767 | 2.36E-15 | 30S ribosomal protein S20 |
| PA1228 | PA1228 | -2.156031 | 0.00067 | hypothetical protein |
| PA2486 | PA2486 | -2.157240 | 0.00145 | hypothetical protein |
| PA0667 | PA0667 | -2.162201 | 2.74E-22 | conserved hypothetical protein |
| PA3598 | PA3598 | -2.162246 | 4.92E-25 | conserved hypothetical protein |
| PA0626 | PA0626 | -2.165966 | 2.17E-10 | hypothetical protein |
| PA2359 | PA2359 | -2.166807 | 2.39E-12 | probable transcriptional regulator |
| PA2137 | PA2137 | -2.171745 | 0.00389 | hypothetical protein |
| PA0913 | *mgtE* | -2.173527 | 6.92E-30 | probable Mg transporter MgtE |
| PA0622 | PA0622 | -2.173544 | 1.03E-10 | probable bacteriophage protein |
| PA5469 | PA5469 | -2.176592 | 2.55E-09 | conserved hypothetical protein |
| PA2246 | *bkdR* | -2.177830 | 3.09E-30 | transcriptional regulator BkdR |
| PA5072 | PA5072 | -2.178558 | 5.18E-20 | probable chemotaxis transducer |
| PA4031 | *ppa* | -2.180163 | 1.56E-15 | inorganic pyrophosphatase |
| PA0497 | PA0497 | -2.180864 | 1.53E-14 | hypothetical protein |
| PA2885 | *atuR* | -2.181652 | 4.33E-21 | putative repressor of atu genes |
| PA3243 | *minC* | -2.183961 | 1.33E-22 | cell division inhibitor MinC |
| PA4738 | PA4738 | -2.188598 | 7.95E-10 | conserved hypothetical protein |
| PA5355 | *glcD* | -2.190236 | 3.80E-19 | glycolate oxidase subunit GlcD |
| PA0620 | PA0620 | -2.199785 | 1.06E-14 | probable bacteriophage protein |
| PA2283 | PA2283 | -2.200478 | 1.70E-10 | hypothetical protein |
| PA1896 | PA1896 | -2.201968 | 1.64E-08 | hypothetical protein |
| PA4639 | PA4639 | -2.203594 | 1.43E-36 | hypothetical protein |
| PA1773 | *cmaX* | -2.203595 | 8.27E-20 | CmaX protein |
| PA0137 | PA0137 | -2.203632 | 0.00047 | probable permease of ABC transporter |
| PA1571 | PA1571 | -2.206629 | 1.95E-15 | hypothetical protein |
| PA4825 | *mgtA* | -2.208154 | 1.19E-16 | Mg(2+) transport ATPase P-type 2 |
| PA1673 | PA1673 | -2.210029 | 4.99E-22 | hypothetical protein |
| PA5481 | PA5481 | -2.214323 | 3.44E-24 | hypothetical protein |
| PA4038 | PA4038 | -2.236327 | 0.01910 | hypothetical protein |
| PA3616 | PA3616 | -2.240438 | 2.65E-20 | conserved hypothetical protein |
| PA0993 | *cupC2* | -2.242326 | 4.67E-07 | chaperone CupC2 |
| PA4378 | *inaA* | -2.243032 | 1.28E-32 | InaA protein |
| PA3370 | PA3370 | -2.243304 | 4.95E-09 | hypothetical protein |
| PA4819 | PA4819 | -2.247323 | 0.00122 | probable glycosyl transferase |
| PA2457 | PA2457 | -2.249315 | 4.67E-10 | hypothetical protein |
| PA5171 | *arcA* | -2.253986 | 4.11E-24 | arginine deiminase |
| PA1891 | PA1891 | -2.262212 | 9.33E-05 | hypothetical protein |
| PA0627 | PA0627 | -2.270585 | 0.00026 | conserved hypothetical protein |
| PA1286 | PA1286 | -2.271791 | 5.88E-06 | probable major facilitator superfamily (MFS) transporter |
| PA3282 | PA3282 | -2.272895 | 5.42E-07 | hypothetical protein |
| PA1029 | PA1029 | -2.278111 | 1.27E-05 | hypothetical protein |
| PA1797 | PA1797 | -2.286618 | 2.71E-14 | hypothetical protein |
| PA4085 | *cupB2* | -2.287746 | 7.68E-06 | chaperone CupB2 |
| PA2357 | *msuE* | -2.292813 | 0.14981 | NADH-dependent FMN reductase MsuE |
| PA0448 | PA0448 | -2.295346 | 1.63E-17 | probable transcriptional regulator |
| PA1180 | phoQ | -2.298827 | 2.09E-19 | two-component sensor PhoQ |
| PA4985 | PA4985 | -2.300946 | 1.29E-15 | hypothetical protein |
| PA4193 | PA4193 | -2.302760 | 0.00033 | probable permease of ABC transporter |
| PA0638 | PA0638 | -2.306471 | 7.06E-09 | probable bacteriophage protein |
| PA5410 | *gbcA* | -2.307347 | 8.05E-10 | GbcA |
| PA1892 | PA1892 | -2.307965 | 2.32E-05 | hypothetical protein |
| PA4167 | PA4167 | -2.322700 | 2.69E-08 | probable oxidoreductase |
| PA1955 | PA1955 | -2.323715 | 0.38512 | hypothetical protein |
| PA4287 | PA4287 | -2.328665 | 2.35E-06 | hypothetical protein |
| PA0990 | PA0990 | -2.330157 | 4.79E-16 | conserved hypothetical protein |
| PA2208 | PA2208 | -2.334064 | 0.06867 | hypothetical protein |
| PA4832 | PA4832 | -2.334429 | 4.65E-07 | probable short-chain dehydrogenase |
| PA4911 | PA4911 | -2.336648 | 0.00010 | probable permease of ABC branched-chain amino acid transporter |
| PA0986 | PA0986 | -2.351925 | 5.01E-14 | conserved hypothetical protein |
| PA0202 | PA0202 | -2.359417 | 1.78E-09 | probable amidase |
| PA0277 | PA0277 | -2.365963 | 4.48E-12 | conserved hypothetical protein |
| PA4826 | PA4826 | -2.370748 | 2.32E-14 | hypothetical protein |
| PA5483 | *algB* | -2.377230 | 6.97E-30 | two-component response regulator AlgB |
| PA0629 | PA0629 | -2.380906 | 3.20E-09 | conserved hypothetical protein |
| PA0293 | *aguB* | -2.388777 | 3.69E-05 | N-carbamoylputrescine amidohydrolase |
| PA3897 | PA3897 | -2.396461 | 2.91E-12 | hypothetical protein |
| PA3444 | PA3444 | -2.399389 | 1.89E-13 | conserved hypothetical protein |
| PA0625 | PA0625 | -2.400637 | 4.08E-05 | hypothetical protein |
| PA2358 | PA2358 | -2.403521 | 3.64E-07 | hypothetical protein |
| PA5172 | *arcB* | -2.414213 | 1.36E-35 | ornithine carbamoyltransferase catabolic |
| PA5180 | PA5180 | -2.421729 | 1.36E-32 | conserved hypothetical protein |
| PA2452 | PA2452 | -2.422737 | 7.63E-28 | hypothetical protein |
| PA1507 | PA1507 | -2.426497 | 2.12E-37 | probable transporter |
| PA0570 | PA0570 | -2.428496 | 0.02659 | hypothetical protein |
| PA1347 | PA1347 | -2.428951 | 2.73E-09 | probable transcriptional regulator |
| PA0985 | *pyoS5* | -2.429563 | 1.29E-16 | pyocin S5 |
| PA1301 | PA1301 | -2.431107 | 1.92E-10 | probable transmembrane sensor |
| PA4087 | PA4087 | -2.432594 | 9.06E-18 | conserved hypothetical protein |
| PA4343 | PA4343 | -2.433502 | 2.48E-07 | probable major facilitator superfamily (MFS) transporter |
| PA0254 | PA0254 | -2.434980 | 1.20E-14 | conserved hypothetical protein |
| PA0619 | PA0619 | -2.437464 | 6.25E-14 | probable bacteriophage protein |
| PA0819 | PA0819 | -2.441451 | 1.88E-07 | hypothetical protein |
| PA1873 | PA1873 | -2.446800 | 8.77E-16 | hypothetical protein |
| PA0623 | PA0623 | -2.449761 | 1.55E-12 | probable bacteriophage protein |
| PA0204 | PA0204 | -2.455494 | 2.31E-08 | probable permease of ABC transporter |
| PA1015 | PA1015 | -2.472197 | 1.31E-18 | probable transcriptional regulator |
| PA0464 | *creC* | -2.473326 | 1.36E-23 | two-component sensor CreC |
| PA0489 | PA0489 | -2.476982 | 1.10E-09 | probable phosphoribosyl transferase |
| PA0807 | *ampDh3* | -2.480252 | 2.99E-21 | AmpDh3 |
| PA0643 | PA0643 | -2.486167 | 3.71E-14 | hypothetical protein |
| PA1845 | PA1845 | -2.489732 | 0.16096 | hypothetical protein |
| PA3575 | PA3575 | -2.492227 | 2.21E-18 | hypothetical protein |
| PA4920 | *nadE* | -2.496679 | 1.81E-23 | NH3-dependent NAD synthetase |
| PA0463 | *creB* | -2.505922 | 2.14E-19 | two-component response regulator CreB |
| PA0621 | PA0621 | -2.512314 | 9.11E-14 | conserved hypothetical protein |
| PA2433 | PA2433 | -2.514786 | 1.79E-11 | hypothetical protein |
| PA3205 | PA3205 | -2.515158 | 2.00E-20 | hypothetical protein |
| PA1300 | PA1300 | -2.516895 | 5.23E-08 | probable sigma-70 factor ECF subfamily |
| PA1988 | *pqqD* | -2.520124 | 0.32622 | pyrroloquinoline quinone biosynthesis protein D |
| PA5538 | *amiA* | -2.521553 | 3.10E-13 | N-acetylmuramoyl-L-alanine amidase |
| PA5328 | PA5328 | -2.524184 | 5.02E-16 | probable cytochrome c(mono-heme type) |
| PA0825 | PA0825 | -2.532560 | 1.26E-05 | hypothetical protein |
| PA4916 | PA4916 | -2.532623 | 2.24E-24 | hypothetical protein |
| PA2825 | *ospR* | -2.535289 | 2.42E-12 | OspR |
| PA5351 | *rubA1* | -2.542095 | 0.06570 | Rubredoxin 1 |
| PA3442 | PA3442 | -2.542317 | 8.45E-07 | probable ATP-binding component of ABC transporter |
| PA0628 | PA0628 | -2.551130 | 2.49E-11 | conserved hypothetical protein |
| PA4629 | PA4629 | -2.570018 | 1.92E-25 | hypothetical protein |
| PA0117 | PA0117 | -2.571232 | 8.68E-15 | probable short chain dehydrogenase |
| PA5470 | PA5470 | -2.575271 | 2.54E-21 | probable peptide chain release factor |
| PA1517 | PA1517 | -2.578109 | 7.33E-28 | conserved hypothetical protein |
| PA1917 | PA1917 | -2.578432 | 0.26528 | hypothetical protein |
| PA1925 | PA1925 | -2.578432 | 0.26528 | hypothetical protein |
| PA3496 | PA3496 | -2.580135 | 2.11E-19 | hypothetical protein |
| PA0617 | PA0617 | -2.585471 | 6.94E-19 | probable bacteriophage protein |
| PA3371 | PA3371 | -2.591431 | 1.21E-06 | hypothetical protein |
| PA2485 | PA2485 | -2.591687 | 3.56E-31 | hypothetical protein |
| PA1179 | *phoP* | -2.592005 | 3.68E-25 | two-component response regulator PhoP |
| PA0639 | PA0639 | -2.593785 | 7.83E-11 | conserved hypothetical protein |
| PA0636 | PA0636 | -2.599939 | 4.25E-15 | hypothetical protein |
| PA2749 | *endA* | -2.605608 | 3.83E-14 | DNA-specific endonuclease I |
| PA3390 | PA3390 | -2.611148 | 1.73E-10 | hypothetical protein |
| PA0630 | PA0630 | -2.614905 | 3.55E-07 | hypothetical protein |
| PA4674 | PA4674 | -2.620277 | 1.81E-32 | conserved hypothetical protein |
| PA0633 | PA0633 | -2.620450 | 1.67E-17 | hypothetical protein |
| PA0641 | PA0641 | -2.625268 | 5.98E-06 | probable bacteriophage protein |
| PA0634 | PA0634 | -2.631433 | 1.47E-17 | hypothetical protein |
| PA4585 | *rtcA* | -2.635106 | 1.04E-17 | RNA 3'-terminal phosphate cyclase |
| PA1435 | PA1435 | -2.646079 | 3.53E-33 | probable Resistance-Nodulation-Cell Division (RND) efflux membrane fusion protein precursor |
| PA4154 | PA4154 | -2.646811 | 1.64E-25 | conserved hypothetical protein |
| PA0637 | PA0637 | -2.654025 | 2.72E-11 | conserved hypothetical protein |
| PA5314 | PA5314 | -2.658183 | 7.98E-18 | hypothetical protein |
| PA4156 | PA4156 | -2.658476 | 2.86E-14 | probable TonB-dependent receptor |
| PA3962 | PA3962 | -2.661303 | 6.33E-29 | hypothetical protein |
| PA0826 | PA0826 | -2.669223 | 1.05E-22 | hypothetical protein |
| PA2415 | PA2415 | -2.673846 | 5.71E-20 | hypothetical protein |
| PA1419 | PA1419 | -2.676621 | 5.03E-11 | probable transporter |
| PA2321 | PA2321 | -2.678198 | 2.02E-24 | gluconokinase |
| PA3480 | PA3480 | -2.681942 | 2.36E-27 | probable deoxycytidine triphosphate deaminase |
| PA3758 | PA3758 | -2.683374 | 6.04E-06 | probable N-acetylglucosamine-6-phosphate deacetylase |
| PA0466 | PA0466 | -2.684436 | 0.27758 | hypothetical protein |
| PA1384 | *galE* | -2.685291 | 2.76E-13 | UDP-glucose 4-epimerase |
| PA3888 | PA3888 | -2.692346 | 6.18E-34 | probable permease of ABC transporter |
| PA2780 | PA2780 | -2.695003 | 3.44E-36 | hypothetical protein |
| PA1898 | *qscR* | -2.701829 | 2.41E-22 | quorum-sensing control repressor |
| PA3540 | *algD* | -2.712763 | 2.19E-20 | GDP-mannose 6-dehydrogenase AlgD |
| PA3443 | PA3443 | -2.714777 | 1.32E-06 | probable permease of ABC transporter |
| PA2145 | PA2145 | -2.717847 | 2.86E-11 | hypothetical protein |
| PA1077 | *flgB* | -2.734207 | 1.02E-24 | flagellar basal-body rod protein FlgB |
| PA1290 | PA1290 | -2.748237 | 5.75E-15 | probable transcriptional regulator |
| PA0635 | PA0635 | -2.751435 | 3.26E-17 | hypothetical protein |
| PA0205 | PA0205 | -2.757830 | 1.62E-06 | probable permease of ABC transporter |
| PA2784 | PA2784 | -2.759786 | 0.00210 | hypothetical protein |
| PA2456 | PA2456 | -2.766246 | 2.69E-20 | hypothetical protein |
| PA0441 | *dht* | -2.768449 | 4.62E-24 | dihydropyrimidinase |
| PA2334 | PA2334 | -2.777859 | 7.72E-12 | probable transcriptional regulator |
| PA4635 | PA4635 | -2.782914 | 3.49E-27 | conserved hypothetical protein |
| PA4586 | PA4586 | -2.793692 | 0.00051 | hypothetical protein |
| PA0640 | PA0640 | -2.794062 | 8.67E-13 | probable bacteriophage protein |
| PA2826 | PA2826 | -2.797863 | 4.31E-32 | probable glutathione peroxidase |
| PA2653 | PA2653 | -2.808051 | 7.98E-28 | probable transporter |
| PA4159 | *fepB* | -2.810721 | 7.91E-06 | ferrienterobactin-binding periplasmic protein precursor FepB |
| PA0618 | PA0618 | -2.814751 | 1.55E-19 | probable bacteriophage protein |
| PA0642 | PA0642 | -2.818279 | 1.44E-05 | hypothetical protein |
| PA2528 | PA2528 | -2.829562 | 8.27E-33 | probable Resistance-Nodulation-Cell Division (RND) efflux membrane fusion protein precursor |
| PA0283 | *sbp* | -2.830341 | 5.65E-23 | sulfate-binding protein precursor |
| PA0684 | PA0684 | -2.834219 | 0.03365 | probable type II secretion system protein |
| PA2258 | *ptxR* | -2.841371 | 7.85E-40 | transcriptional regulator PtxR |
| PA1417 | PA1417 | -2.841714 | 6.86E-13 | probable decarboxylase |
| PA4881 | PA4881 | -2.855388 | 2.59E-07 | hypothetical protein |
| PA3449 | PA3449 | -2.859333 | 2.01E-07 | conserved hypothetical protein |
| PA0087 | PA0087 | -2.870358 | 8.06E-05 | hypothetical protein |
| PA1298 | PA1298 | -2.872549 | 3.98E-18 | conserved hypothetical protein |
| PA0971 | *tolA* | -2.887241 | 3.05E-33 | TolA protein |
| PA0632 | PA0632 | -2.909820 | 2.52E-05 | hypothetical protein |
| PA5157 | PA5157 | -2.913289 | 4.29E-12 | probable transcriptional regulator |
| PA0161 | PA0161 | -2.916321 | 2.26E-31 | hypothetical protein |
| PA1260 | PA1260 | -2.927177 | 9.32E-06 | amino acid ABC transporter periplasmic binding protein |
| PA2672 | PA2672 | -2.931947 | 0.03181 | probable type II secretion system protein |
| PA0229 | *pcaT* | -2.936782 | 1.34E-36 | dicarboxylic acid transporter PcaT |
| PA0805 | PA0805 | -2.938972 | 2.09E-30 | hypothetical protein |
| PA5403 | PA5403 | -2.939316 | 1.39E-05 | probable transcriptional regulator |
| PA4095 | PA4095 | -2.959597 | 0.02761 | hypothetical protein |
| PA2668 | PA2668 | -2.982468 | 0.02600 | hypothetical protein |
| PA4912 | PA4912 | -2.985524 | 4.63E-13 | branched chain amino acid ABC transporter membrane protein |
| PA1418 | PA1418 | -2.985895 | 2.61E-13 | probable sodium:solute symport protein |
| PA3133 | PA3133 | -2.988619 | 1.30E-11 | probable transcriptional regulator |
| PA0680 | PA0680 | -2.992586 | 0.07654 | HxcV putative pseudopilin |
| PA2569 | PA2569 | -2.993291 | 1.94E-22 | hypothetical protein |
| PA1952 | PA1952 | -2.993712 | 0.24582 | hypothetical protein |
| PA3730 | PA3730 | -2.994065 | 3.56E-22 | hypothetical protein |
| PA2285 | PA2285 | -2.999877 | 0.00632 | hypothetical protein |
| PA2792 | PA2792 | -3.022332 | 1.25E-10 | hypothetical protein |
| PA1299 | PA1299 | -3.030529 | 1.77E-22 | conserved hypothetical protein |
| PA5130 | PA5130 | -3.036823 | 5.96E-23 | conserved hypothetical protein |
| PA5417 | *soxD* | -3.038465 | 0.06850 | sarcosine oxidase delta subunit |
| PA4649 | PA4649 | -3.039325 | 0.00941 | hypothetical protein |
| PA2310 | PA2310 | -3.050683 | 8.45E-11 | hypothetical protein |
| PA0616 | PA0616 | -3.066101 | 2.31E-22 | hypothetical protein |
| PA4122 | PA4122 | -3.066197 | 6.86E-07 | conserved hypothetical protein |
| PA1924 | PA1924 | -3.070749 | 0.18010 | hypothetical protein |
| PA1416 | PA1416 | -3.072049 | 1.91E-17 | conserved hypothetical protein |
| PA5402 | PA5402 | -3.076022 | 1.59E-16 | hypothetical protein |
| PA4763 | *recN* | -3.118004 | 4.59E-42 | DNA repair protein RecN |
| PA4584 | PA4584 | -3.118116 | 1.32E-31 | conserved hypothetical protein |
| PA0910 | PA0910 | -3.128352 | 1.61E-13 | hypothetical protein |
| PA1502 | *gcl* | -3.153976 | 4.66E-16 | glyoxylate carboligase |
| PA5170 | *arcD* | -3.155615 | 5.69E-61 | arginine/ornithine antiporter |
| PA3283 | PA3283 | -3.162357 | 1.96E-11 | conserved hypothetical protein |
| PA4917 | PA4917 | -3.170366 | 3.19E-35 | hypothetical protein |
| PA0980 | PA0980 | -3.210199 | 1.13E-30 | hypothetical protein |
| PA4921 | PA4921 | -3.221816 | 1.69E-32 | hypothetical protein |
| PA4785 | PA4785 | -3.239182 | 8.57E-46 | probable acyl-CoA thiolase |
| PA3389 | PA3389 | -3.258001 | 1.86E-08 | probable ring-cleaving dioxygenase |
| PA4731 | *panD* | -3.259828 | 1.09E-16 | aspartate 1-decarboxylase precursor |
| PA2284 | PA2284 | -3.277885 | 1.70E-10 | hypothetical protein |
| PA0191 | PA0191 | -3.284375 | 7.78E-27 | probable transcriptional regulator |
| PA2766 | PA2766 | -3.295063 | 8.98E-19 | probable transcriptional regulator |
| PA1178 | *oprH* | -3.340048 | 6.55E-45 | PhoP/Q and low Mg2+ inducible outer membrane protein H1 precursor |
| PA0197 | *tonB2* | -3.369662 | 4.57E-07 | TonB2 |
| PA0848 | PA0848 | -3.372689 | 4.88E-49 | probable alkyl hydroperoxide reductase |
| PA3661 | PA3661 | -3.386689 | 0.00028 | hypothetical protein |
| PA3447 | PA3447 | -3.396504 | 0.00153 | probable ATP-binding component of ABC transporter |
| PA1343 | PA1343 | -3.406110 | 5.56E-19 | hypothetical protein |
| PA2429 | PA2429 | -3.439873 | 0.00092 | hypothetical protein |
| PA2432 | *bexR* | -3.459703 | 1.36E-35 | bistable expression regulator BexR |
| PA2667 | PA2667 | -3.465528 | 1.88E-61 | conserved hypothetical protein |
| PA0203 | PA0203 | -3.474901 | 2.06E-07 | probable binding protein component of ABC transporter |
| PA2286 | PA2286 | -3.492320 | 1.61E-16 | hypothetical protein |
| PA0907 | PA0907 | -3.493748 | 1.04E-54 | hypothetical protein |
| PA2312 | PA2312 | -3.551778 | 2.21E-17 | probable transcriptional regulator |
| PA1297 | PA1297 | -3.597233 | 2.45E-37 | probable metal transporter |
| PA0614 | PA0614 | -3.602733 | 2.44E-37 | hypothetical protein |
| PA1329 | PA1329 | -3.614774 | 0.00333 | conserved hypothetical protein |
| PA0911 | PA0911 | -3.636128 | 3.08E-17 | hypothetical protein |
| PA4583 | PA4583 | -3.641370 | 6.27E-47 | conserved hypothetical protein |
| PA0922 | PA0922 | -3.653332 | 2.02E-53 | hypothetical protein |
| PA0874 | PA0874 | -3.687433 | 2.68E-40 | hypothetical protein |
| PA1977 | PA1977 | -3.689733 | 0.04568 | hypothetical protein |
| PA1897 | PA1897 | -3.694099 | 3.72E-21 | hypothetical protein |
| PA3552 | *arnB* | -3.695877 | 1.13E-32 | ArnB |
| PA0124 | PA0124 | -3.698319 | 2.28E-40 | hypothetical protein |
| PA3007 | *lexA* | -3.702617 | 6.51E-60 | repressor protein LexA |
| PA3450 | PA3450 | -3.769125 | 1.89E-38 | probable antioxidant protein |
| PA3937 | PA3937 | -3.783198 | 3.06E-20 | probable ATP-binding component of ABC taurine transporter |
| PA5471 | PA5471 | -3.789453 | 7.26E-40 | hypothetical protein |
| PA4195 | PA4195 | -3.817454 | 3.08E-28 | probable binding protein component of ABC transporter |
| PA0182 | PA0182 | -3.832563 | 3.58E-32 | probable short-chain dehydrogenase |
| PA4739 | PA4739 | -3.872880 | 1.28E-37 | conserved hypothetical protein |
| PA1983 | *exaB* | -3.884824 | 0.02963 | cytochrome c550 |
| PA0671 | PA0671 | -3.890419 | 1.05E-31 | hypothetical protein |
| PA2287 | PA2287 | -3.890747 | 1.15E-29 | hypothetical protein |
| PA0284 | PA0284 | -3.909089 | 2.14E-24 | hypothetical protein |
| PA1911 | *femR* | -3.928101 | 0.02222 | sigma factor regulator FemR |
| PA5471 | PA5471 | -3.934213 | 1.65E-27 | leader peptide |
| PA4354 | PA4354 | -3.986433 | 2.61E-08 | conserved hypothetical protein |
| PA1856 | PA1856 | -4.015937 | 4.35E-48 | probable cytochrome oxidase subunit |
| PA1980 | *eraR* | -4.018168 | 0.01880 | response regulator EraR |
| PA0424 | *mexR* | -4.051786 | 1.02E-82 | multidrug resistance operon repressor MexR |
| PA3757 | PA3757 | -4.064803 | 4.82E-16 | probable transcriptional regulator |
| PA2288 | PA2288 | -4.082206 | 4.82E-50 | hypothetical protein |
| PA0125 | PA0125 | -4.134344 | 5.01E-34 | hypothetical protein |
| PA5482 | PA5482 | -4.135483 | 7.34E-51 | hypothetical protein |
| PA1291 | PA1291 | -4.179576 | 6.72E-41 | hypothetical protein |
| PA2311 | PA2311 | -4.183947 | 4.72E-10 | hypothetical protein |
| PA1919 | *nrdG* | -4.236368 | 0.22528 | class III (anaerobic) ribonucleoside-triphosphate reductase activating protein activase NrdG |
| PA3446 | PA3446 | -4.256432 | 1.11E-58 | conserved hypothetical protein |
| PA3990 | PA3990 | -4.267439 | 4.85E-19 | conserved hypothetical protein |
| PA3369 | PA3369 | -4.290842 | 2.09E-39 | hypothetical protein |
| PA4582 | PA4582 | -4.314226 | 1.51E-92 | conserved hypothetical protein |
| PA0908 | PA0908 | -4.331304 | 3.32E-10 | hypothetical protein |
| PA2768 | PA2768 | -4.466458 | 0.00418 | hypothetical protein |
| PA1953 | PA1953 | -4.536066 | 0.00439 | hypothetical protein |
| PA4121 | PA4121 | -4.571684 | 8.05E-05 | conserved hypothetical protein |
| PA0610 | *prtN* | -4.609785 | 1.90E-14 | transcriptional regulator PrtN |
| PA0909 | PA0909 | -4.694454 | 0.00055 | hypothetical protein |
| PA1974 | PA1974 | -4.810112 | 0.18485 | hypothetical protein |
| PA1855 | PA1855 | -4.830950 | 6.07E-10 | hypothetical protein |
| PA3445 | PA3445 | -4.842319 | 5.41E-51 | conserved hypothetical protein |
| PA1986 | *pqqB* | -4.866348 | 0.00129 | pyrroloquinoline quinone biosynthesis protein B |
| PA1987 | *pqqC* | -4.983062 | 0.00103 | pyrroloquinoline quinone biosynthesis protein C |
| PA2006 | PA2006 | -5.008892 | 0.00102 | probable major facilitator superfamily (MFS) transporter |
| PA1940 | PA1940 | -5.010420 | 0.00061 | hypothetical protein |
| PA3938 | PA3938 | -5.013980 | 1.54E-70 | probable periplasmic taurine-binding protein precursor |
| PA1922 | PA1922 | -5.132134 | 0.00036 | probable TonB-dependent receptor |
| PA1909 | PA1909 | -5.141759 | 0.00042 | hypothetical protein |
| PA2021 | PA2021 | -5.245206 | 0.00032 | hypothetical protein |
| PA2022 | PA2022 | -5.308181 | 0.00022 | probable nucleotide sugar dehydrogenase |
| PA1942 | PA1942 | -5.356020 | 0.00017 | hypothetical protein |
| PA1912 | *femI* | -5.377259 | 0.00014 | ECF sigma factor FemI |
| PA4919 | *pncB1* | -5.388408 | 2.25E-84 | nicotinate phosphoribosyltransferase |
| PA1938 | PA1938 | -5.391059 | 4.31E-26 | conserved hypothetical protein |
| PA0612 | *ptrB* | -5.434996 | 3.16E-12 | repressor PtrB |
| PA1916 | PA1916 | -5.510330 | 9.43E-05 | probable amino acid permease |
| PA2026 | PA2026 | -5.515301 | 0.00016 | conserved hypothetical protein |
| PA1954 | PA1954 | -5.550252 | 7.75E-05 | hypothetical protein |
| PA1918 | PA1918 | -5.584761 | 5.24E-05 | hypothetical protein |
| PA0613 | PA0613 | -5.597265 | 1.63E-10 | hypothetical protein |
| PA1957 | PA1957 | -5.656497 | 6.01E-05 | hypothetical protein |
| PA1956 | PA1956 | -5.679397 | 3.41E-05 | hypothetical protein |
| PA0729 | PA0729 | -5.708637 | 1.47E-13 | hypothetical protein |
| PA2925 | *hisM* | -5.765205 | 2.83E-05 | histidine transport system permease HisM |
| PA1914 | PA1914 | -5.933332 | 9.23E-06 | conserved hypothetical protein |
| PA2007 | *maiA* | -5.957826 | 8.84E-06 | maleylacetoacetate isomerase |
| PA1978 | *erbR* | -6.030569 | 5.67E-06 | response regulator ErbR |
| PA1937 | PA1937 | -6.095730 | 5.96E-06 | conserved hypothetical protein |
| PA2002 | PA2002 | -6.104583 | 8.37E-06 | conserved hypothetical protein |
| PA1975 | PA1975 | -6.127376 | 4.87E-06 | hypothetical protein |
| PA1972 | PA1972 | -6.139046 | 4.63E-06 | conserved hypothetical protein |
| PA2004 | PA2004 | -6.215400 | 3.31E-06 | conserved hypothetical protein |
| PA1996 | *ppiC1* | -6.237997 | 4.32E-06 | peptidyl-prolyl cis-trans isomerase C1 |
| PA4918 | PA4918 | -6.252187 | 1.52E-94 | hypothetical protein |
| PA1910 | *femA* | -6.388149 | 1.29E-06 | ferric-mycobactin receptor FemA |
| PA1990 | *pqqH* | -6.430992 | 1.31E-06 | PqqH |
| PA1982 | *exaA* | -6.487981 | 1.20E-06 | quinoprotein ethanol dehydrogenase |
| PA1967 | PA1967 | -6.503534 | 7.70E-07 | hypothetical protein |
| PA1966 | PA1966 | -6.559375 | 5.15E-07 | hypothetical protein |
| PA5105 | *hutC* | -6.562137 | 1.33E-62 | histidine utilization repressor HutC |
| PA1976 | *ercS'* | -6.626199 | 3.23E-07 | ErcS' |
| PA2000 | *dhcB* | -6.628693 | 6.13E-07 | DhcB dehydrocarnitine CoA transferase subunit B |
| PA2013 | *liuC* | -6.716173 | 2.86E-07 | putative 3-methylglutaconyl-CoA hydratase |
| PA1994 | PA1994 | -6.719841 | 3.66E-07 | hypothetical protein |
| PA1923 | PA1923 | -6.866492 | 2.59E-07 | hypothetical protein |
| PA1973 | *pqqF* | -6.989036 | 5.75E-08 | pyrroloquinoline quinone biosynthesis protein F |
| PA1936 | PA1936 | -7.057299 | 3.06E-08 | hypothetical protein |
| PA1934 | PA1934 | -7.070497 | 3.90E-08 | hypothetical protein |
| PA1989 | *pqqE* | -7.078101 | 2.43E-08 | pyrroloquinoline quinone biosynthesis protein E |
| PA2008 | *fahA* | -7.129754 | 1.85E-08 | fumarylacetoacetase |
| PA1951 | PA1951 | -7.197384 | 1.99E-08 | hypothetical protein |
| PA2003 | *bdhA* | -7.350563 | 6.28E-09 | 3-hydroxybutyrate dehydrogenase |
| PA2011 | *liuE* | -7.403336 | 4.73E-09 | 3-hydroxy-3-methylglutaryl-CoA lyase |
| PA1965 | PA1965 | -7.455130 | 3.82E-09 | hypothetical protein |
| PA1958 | PA1958 | -7.483378 | 2.28E-09 | probable transporter |
| PA1931 | PA1931 | -7.509669 | 1.72E-09 | probable ferredoxin |
| PA2010 | PA2010 | -7.541725 | 1.39E-09 | probable transcriptional regulator |
| PA1992 | *ercS* | -7.577386 | 1.76E-09 | ErcS |
| PA1928 | *rimJ* | -7.632947 | 1.06E-09 | ribosomal protein alanine acetyltransferase |
| PA1993 | PA1993 | -7.653994 | 9.18E-10 | probable major facilitator superfamily (MFS) transporter |
| PA1962 | *azoR2* | -7.657378 | 6.97E-10 | FMN-dependent NADH-azoreductase AzoR2 |
| PA1921 | PA1921 | -7.704363 | 9.52E-10 | hypothetical protein |
| PA1960 | PA1960 | -7.720507 | 5.58E-10 | hypothetical protein |
| PA1995 | PA1995 | -7.721614 | 7.19E-10 | hypothetical protein |
| PA1961 | PA1961 | -7.742825 | 6.37E-10 | probable transcriptional regulator |
| PA2019 | PA2019 | -7.788015 | 9.29E-10 | Resistance-Nodulation-Cell Division (RND) multidrug efflux membrane fusion protein precursor |
| PA2024 | PA2024 | -7.901561 | 2.21E-10 | probable ring-cleaving dioxygenase |
| PA0724 | PA0724 | -7.914776 | 1.17E-24 | probable coat protein A of bacteriophage Pf1 |
| PA1969 | PA1969 | -7.941649 | 2.18E-10 | hypothetical protein |
| PA1971 | *braZ* | -8.030205 | 8.58E-11 | branched chain amino acid transporter BraZ |
| PA1968 | PA1968 | -8.051854 | 7.97E-11 | hypothetical protein |
| PA0727 | PA0727 | -8.070797 | 4.71E-23 | hypothetical protein from bacteriophage Pf1 |
| PA1932 | PA1932 | -8.080371 | 1.59E-10 | probable hydroxylase molybdopterin-containing subunit |
| PA2020 | PA2020 | -8.182187 | 1.05E-10 | probable transcriptional regulator |
| PA2023 | *galU* | -8.406937 | 3.80E-11 | UTP--glucose-1-phosphate uridylyltransferase |
| PA1998 | *dhcR* | -8.491516 | 6.30E-12 | DhcR transcriptional regulator |
| PA1943 | PA1943 | -8.543542 | 2.48E-12 | hypothetical protein |
| PA0728 | PA0728 | -8.545460 | 5.89E-21 | probable bacteriophage integrase |
| PA1997 | PA1997 | -8.579100 | 6.97E-12 | probable AMP-binding enzyme |
| PA1963 | PA1963 | -8.622089 | 2.00E-12 | hypothetical protein |
| PA2012 | *liuD* | -8.629184 | 1.87E-12 | methylcrotonyl-CoA carboxylase alpha-subunit (biotin-containing) |
| PA1991 | PA1991 | -8.632665 | 3.60E-12 | probable iron-containing alcohol dehydrogenase |
| PA2018 | PA2018 | -8.669746 | 1.17E-12 | Resistance-Nodulation-Cell Division (RND) multidrug efflux transporter |
| PA2001 | *atoB* | -8.798235 | 1.38E-12 | acetyl-CoA acetyltransferase |
| PA1913 | PA1913 | -8.835757 | 4.47E-13 | hypothetical protein |
| PA1927 | *metE* | -8.860637 | 4.79E-13 | 5-methyltetrahydropteroyltriglutamate-homocysteine S-methyltransferase |
| PA1999 | *dhcA* | -8.889334 | 1.41E-12 | DhcA%2C dehydrocarnitine CoA transferase subunit A |
| PA0726 | PA0726 | -8.918128 | 1.39E-22 | hypothetical protein of bacteriophage Pf1 |
| PA2005 | PA2005 | -8.959491 | 1.88E-13 | probable transcriptional regulator |
| PA2009 | *hmgA* | -9.037959 | 1.61E-13 | homogentisate 2-dioxygenase |
| PA1945 | PA1945 | -9.129268 | 1.63E-13 | probable transcriptional regulator |
| PA2016 | *liuR* | -9.131902 | 5.97E-14 | regulator of liu genes |
| PA2014 | *liuB* | -9.157956 | 6.72E-14 | methylcrotonyl-CoA carboxylase beta-subunit |
| PA0721 | PA0721 | -9.293291 | 2.04E-14 | hypothetical protein of bacteriophage Pf1 |
| PA1920 | *nrdD* | -9.320093 | 3.29E-14 | class III (anaerobic) ribonucleoside-triphosphate reductase subunit NrdD |
| PA2017 | PA2017 | -9.334070 | 2.08E-14 | hypothetical protein |
| PA1929 | PA1929 | -9.407276 | 9.13E-15 | hypothetical protein |
| PA1985 | *pqqA* | -9.584573 | 5.22E-15 | pyrroloquinoline quinone biosynthesis protein A |
| PA1935 | PA1935 | -9.603632 | 1.60E-15 | hypothetical protein |
| PA0720 | PA0720 | -9.615946 | 0 | helix destabilizing protein of bacteriophage Pf1 |
| PA1959 | *bacA* | -9.653193 | 2.70E-15 | bacitracin resistance protein |
| PA1948 | *rbsC* | -9.670539 | 4.61E-15 | membrane protein component of ABC ribose transporter |
| PA1930 | PA1930 | -9.675800 | 9.30E-16 | probable chemotaxis transducer |
| PA1941 | PA1941 | -9.814894 | 6.32E-16 | hypothetical protein |
| PA1933 | PA1933 | -9.930968 | 2.66E-16 | probable hydroxylase large subunit |
| PA0723 | *coaB* | -10.00999 | 0 | coat protein B of bacteriophage Pf1 |
| PA1915 | PA1915 | -10.03613 | 1.70E-16 | hypothetical protein |
| PA1949 | *rbsR* | -10.28405 | 3.85E-17 | ribose operon repressor RbsR |
| PA1926 | PA1926 | -10.32835 | 1.25E-17 | conserved hypothetical protein |
| PA0725 | PA0725 | -10.42807 | 3.20E-33 | hypothetical protein of bacteriophage Pf1 |
| PA0722 | PA0722 | -10.45030 | 8.40E-12 | hypothetical protein of bacteriophage Pf1 |
| PA1944 | PA1944 | -10.51426 | 4.65E-18 | hypothetical protein |
| PA1964 | PA1964 | -10.98391 | 5.57E-20 | probable ATP-binding component of ABC transporter |
| PA2025 | *gor* | -11.16985 | 1.71E-20 | glutathione reductase |
| PA0718 | PA0718 | -11.54848 | 8.49E-69 | hypothetical protein of bacteriophage Pf1 |
| PA2015 | *liuA* | -11.72131 | 2.09E-22 | putative isovaleryl-CoA dehydrogenase |
| PA1950 | *rbsK* | -11.76263 | 3.24E-22 | ribokinase |
| PA0719 | PA0719 | -11.80871 | 6.41E-16 | hypothetical protein of bacteriophage Pf1 |
| PA1947 | *rbsA* | -11.89838 | 1.55E-22 | ribose transport protein RbsA |
| PA0717 | PA0717 | -12.56362 | 0 | hypothetical protein of bacteriophage Pf1 |
| PA1939 | PA1939 | -12.88697 | 2.95E-27 | hypothetical protein |
| PA1946 | *rbsB* | -14.29008 | 3.23E-32 | binding protein component precursor of ABC ribose transporter |

**Table S2. Differential metabolites between the Δ*hutC* mutant and PAO1 (Δ*hutC* vs PAO1) by untargeted LS-MS metabolomics**

| Metabolite | 代谢物 | *P*value | FDR | VIP | FC | Regulate | KEGG Pathway Description |
| --- | --- | --- | --- | --- | --- | --- | --- |
| Prolylphenylalanine | 脯氨酰苯丙氨酸 | 0.00919 | 0.1752 | 2.60 | 1.22 | up | - |
| 5'-Deoxy-5-Fluorocytidine | - | 0.02043 | 0.2412 | 2.34 | 1.16 | up | Drug metabolism - other enzymes |
| 2-Acetoxybenzonitrile | - | 0.01259 | 0.2037 | 2.34 | 1.15 | up | - |
| Zinostatin | - | 0.00890 | 0.1722 | 2.36 | 1.13 | up | - |
| Lysope | - | 0.00732 | 0.1650 | 2.59 | 1.13 | up | - |
| Mangiferin | 芒果苷 | 0.00430 | 0.1367 | 2.29 | 1.13 | up | - |
| Phenazine-1-Carboxylic Acid | 吩嗪-1-羧酸 | 0.02620 | 0.2585 | 2.15 | 1.12 | up | - |
| Nicotinamide | 烟酰胺 | 0.00262 | 0.1030 | 2.49 | 1.11 | up | Nicotinate and nicotinamide metabolism;Biosynthesis of cofactors |
| N-Docosahexaenoyl Lysine | N -二十二碳六烯酰赖氨酸 | 0.01612 | 0.2236 | 2.20 | 1.11 | up | - |
| 2,4-Quinolinediol | 2，4 -喹啉二醇 | 0.02496 | 0.2569 | 1.79 | 1.09 | up | Biosynthesis of various alkaloids |
| Guanosine 3'-Monophosphate | - | 0.02781 | 0.2638 | 1.81 | 1.08 | up | Metabolic pathways;Purine metabolism |
| 12-Aminododecanoic Acid | - | 0.03801 | 0.2941 | 1.70 | 1.08 | up | - |
| 1-Hydroxyphenazine | 1-羟基吩嗪 | 0.00054 | 0.0573 | 1.89 | 1.08 | up | Biosynthesis of secondary metabolites;Phenazine biosynthesis |
| Adp-Ribose2'-Phosphate | - | 0.00038 | 0.0455 | 2.20 | 1.08 | up | - |
| N-Butanoyl-Dl-HomoserineLactone | - | 0.00096 | 0.0719 | 1.98 | 1.07 | up | - |
| Norketamine | 去甲氯胺酮 | 0.01954 | 0.2369 | 1.63 | 1.07 | up | - |
| N1-(5-Phospho-D-Ribosyl)-Amp | - | 0.01832 | 0.2314 | 1.74 | 1.06 | up | Histidinemetabolism;Biosynthesis of secondary metabolites;Biosynthesis of amino acids |
| 2-Hydroxyhippuric Acid | - | 0.00280 | 0.1068 | 1.57 | 1.06 | up | - |
| 6-Chloro-5-Methyl-N-Quinolin-4-Yl-2,3-Dihydroindole-1-Carboxamide | - | 0.04225 | 0.3053 | 1.62 | 1.06 | up | - |
| Acetaminophen | 对乙酰氨基酚 | 0.00199 | 0.0909 | 1.68 | 1.06 | up | - |
| Buflomedil | 丁咯地尔 | 0.01568 | 0.2227 | 1.54 | 1.06 | up | - |
| N1-Methyl-2-Pyridone-5-Carboxamide | - | 0.00294 | 0.1093 | 1.49 | 1.06 | up | Metabolic pathways;Nicotinate and nicotinamide metabolism |
| (Z)-2-(5-Tetradecenyl)Cyclobutanone | - | 0.04239 | 0.3053 | 1.62 | 1.06 | up | - |
| Enalapril | 苯丁酯脯酸 | 0.03542 | 0.2892 | 1.45 | 1.06 | up | - |
| L-Xylulose | L-木酮糖 | 0.00213 | 0.0925 | 1.79 | 1.05 | up | Metabolic pathways;Pentose and glucuronate interconversions |
| 3-Methylamino-1,2-Propandiol | - | 0.03998 | 0.2986 | 1.42 | 1.05 | up | - |
| 1-Heptadecanoyl-Glycero-3-Phosphate | - | 0.00056 | 0.0576 | 1.84 | 1.05 | up | - |
| D-Phenylalanyl-L-2-Piperidinecarbonyl-N-(4-Nitro Phenyl)-L-Argininamide | - | 0.00213 | 0.0925 | 1.63 | 1.05 | up | - |
| Adenosine Diphosphate Ribose | - | 0.02807 | 0.2639 | 1.64 | 1.05 | up | Metabolic pathways;Purine metabolism |
| Triphenylphosphine Oxide | 三苯基氧膦 | 0.00218 | 0.0939 | 1.57 | 1.05 | up | - |
| Lexipafant | 来昔帕泛 | 0.00478 | 0.1421 | 1.76 | 1.05 | up | - |
| 1-Hexadecanoyl-Sn-Glycero-3-Phosphoethanolamine | - | 0.00146 | 0.0844 | 1.87 | 1.05 | up | - |
| Geranic Acid | 牻牛儿酸 | 0.00632 | 0.1585 | 1.48 | 1.05 | up | Pinene, camphor and geraniol degradation; Microbial metabolism in diverse environments |
| Pe(17:1/0:0) | - | 0.00743 | 0.1654 | 1.75 | 1.05 | up | - |
| 2-Aminoacetophenone | 2’-氨基苯乙酮 | 0.03127 | 0.2761 | 1.41 | 1.05 | up | - |
| Lysopc(18:1(11Z)/0:0) | - | 0.04287 | 0.3053 | 1.34 | 1.05 | up | Glycerophospholipid metabolism |
| Ethyl 2-(5,7-Dihydroxy-2-Oxochromen-4-Yl)Acetate | - | 0.04504 | 0.3100 | 1.37 | 1.05 | up | - |
| Mg(0:0/20:5(5Z,8Z,11Z,14Z,17Z)/0:0) | - | 0.04068 | 0.2994 | 1.38 | 1.04 | up | - |
| 4-Methyl-5-(2-Phosphonooxyethyl)Thiazole | - | 0.00026 | 0.0381 | 1.41 | 1.04 | up | Thiamine metabolism; Metabolic pathways; Biosynthesis of cofactors |
| N-Acetylanthranilic Acid | - | 0.00490 | 0.1434 | 1.33 | 1.04 | up | Biosynthesis of various alkaloids |
| Adenine | 腺嘌呤 | 0.04799 | 0.3189 | 1.25 | 1.04 | up | Metabolic pathways; Biosynthesis of secondary metabolites;Nucleotide metabolism;Purine metabolism |
| Physalolactone | 酸浆内酯 | 3.57E-05 | 0.0275 | 1.59 | 1.04 | up | - |
| Trimethylamine N-Oxide | 三甲胺N-氧化物 | 0.00417 | 0.1335 | 1.31 | 1.04 | up | Methane metabolism; Metabolic pathways; Microbial metabolism in diverse environments; Two-component system |
| 1-Palmitoyl-Dihydroxyacetone-Phosphate | - | 0.03296 | 0.2798 | 1.31 | 1.04 | up | - |
| 5-Phenylvaleric Acid | - | 0.02904 | 0.2678 | 1.17 | 1.04 | up | - |
| Pe(16:1/0:0) | - | 0.02994 | 0.2705 | 1.48 | 1.04 | up | - |
| 12-Hydroxyjasmonic Acid Glucoside | - | 0.00236 | 0.0979 | 1.35 | 1.04 | up | - |
| Peridinin | 多甲藻(黄)素 | 0.00371 | 0.1253 | 1.51 | 1.04 | up | - |
| 2-Hydroxyquinoline | 2-羟基喹啉 | 0.03250 | 0.2793 | 1.15 | 1.04 | up | - |
| Vignatic Acid A | - | 0.00330 | 0.1165 | 1.47 | 1.04 | up | - |
| Cytosine | 胞嘧啶 | 0.01695 | 0.2278 | 1.28 | 1.04 | up | Pyrimidine metabolism; Metabolic pathways; Nucleotide metabolism |
| N-Acetylcytidine | - | 0.02515 | 0.2570 | 1.32 | 1.04 | up | - |
| Lys Met His | 赖氨酸 甲硫氨酸 组氨酸 | 0.04085 | 0.2997 | 1.45 | 1.04 | up | - |
| 1-[3,4-Dihydroxy-5-(Hydroxymethyl)Oxolan-2-Yl]-4-Hydroxyhydropyridin-2-One | - | 0.01203 | 0.1993 | 1.44 | 1.04 | up | - |
| Lpe(16:0) | - | 0.02237 | 0.2502 | 1.50 | 1.04 | up | - |
| 4-Butylmorpholine | 4 -丁基吗啉 | 0.01057 | 0.1887 | 1.21 | 1.04 | up | - |
| Oleamide | 油酸酰胺 | 0.03735 | 0.2941 | 1.47 | 1.04 | up | - |
| Udp-N-Acetyl-D-Galactosamine | - | 0.00059 | 0.0589 | 1.48 | 1.04 | up | Metabolic pathways;Amino sugar and nucleotide sugar metabolism;Biosynthesis of nucleotide sugars;O-Antigen nucleotide sugar biosynthesis; Teichoic acid biosynthesis |
| Benzamide | 苯甲酰胺 | 0.04276 | 0.3053 | 1.08 | 1.04 | up | Aminobenzoate degradation; Microbial metabolism in diverse environments |
| Prolyl-Gamma-Glutamate | - | 0.02613 | 0.2585 | 1.18 | 1.03 | up | - |
| 2-Aminopurine | 2-氨基嘌呤 | 0.02807 | 0.2639 | 1.22 | 1.03 | up | - |
| 4-Methylene-2-Octyl-5-Oxotetrahydrofuran-3-Carboxylic Acid | - | 0.02200 | 0.2490 | 1.15 | 1.03 | up | - |
| 2-Pyrrolidinone | 2-吡咯烷酮 | 0.03853 | 0.2956 | 1.15 | 1.03 | up | - |
| Pro Gln | - | 0.01436 | 0.2135 | 1.30 | 1.03 | up | - |
| N-Jasmonoylisoleucine | N -茉莉酸基异亮氨酸 | 0.01290 | 0.2067 | 1.11 | 1.03 | up | - |
| Benzothiazole | 苯并噻唑 | 0.04742 | 0.3169 | 1.03 | 1.03 | up | - |
| 3-Acetamidophenol | - | 0.00854 | 0.1698 | 1.13 | 1.03 | up | - |
| Zidovudine Diphosphate | - | 0.02141 | 0.2460 | 1.21 | 1.03 | up | - |
| 20-Oxo-Leukotriene E4 | - | 0.03141 | 0.2761 | 1.26 | 1.03 | up | - |
| Pe(19:1(9Z)/0:0) | - | 0.03077 | 0.2732 | 1.28 | 1.03 | up | - |
| 4-Methylphthalic Anhydride | - | 0.02620 | 0.2585 | 1.10 | 1.03 | up | - |
| Uridine Diphosphate-N-Acetylgalactosamine | - | 0.00489 | 0.1434 | 1.40 | 1.03 | up | - |
| Choline | 胆碱 | 0.00135 | 0.0833 | 1.32 | 1.03 | up | Metabolic pathways; Glycerophospholipid metabolism;ABC transporters; Teichoic acid biosynthesis;Glycine, serine and threonine metabolism |
| Umbelliferone | 伞形酮 | 0.04834 | 0.3189 | 1.04 | 1.03 | up | Biosynthesis of secondary metabolites;Biosynthesis of various plant secondary metabolites |
| 5'-Deoxy-5'-Methylthioadenosine | 5 ' -脱氧- 5 ' -甲硫腺苷 | 0.01641 | 0.2257 | 1.21 | 1.03 | up | Metabolic pathways; Cysteine and methionine metabolism |
| Phyllanthusol B | 叶醇B | 0.00302 | 0.1110 | 1.24 | 1.03 | up | - |
| Cyclohexane | 环己烷 | 0.03037 | 0.2724 | 1.33 | 1.03 | up | Caprolactam degradation; Microbial metabolism in diverse environments |
| Coniferyl Alcohol | 松柏醇 | 0.03254 | 0.2793 | 1.04 | 1.03 | up | Metabolic pathways; Biosynthesis of secondary metabolites;Biosynthesis of various plant secondary metabolites |
| Isocytosine | - | 0.00810 | 0.1691 | 1.17 | 1.03 | up | - |
| Hydroxysuberic Acid | - | 0.01408 | 0.2115 | 1.12 | 1.03 | up | - |
| Glycylprolylhydroxyproline | 甘氨酰脯氨酰羟基脯氨酸 | 8.37E-05 | 0.0275 | 1.28 | 1.03 | up | - |
| 3-(3,4-Dihydroxyphenyl)Prop-2-Enoic Acid | - | 0.02675 | 0.2585 | 1.03 | 1.03 | up | - |
| Pyocyanine | 绿脓菌素 | 0.00252 | 0.1015 | 1.35 | 1.03 | up | Metabolic pathways; Biosynthesis of secondary metabolites;Phenazine biosynthesis |
| 20-Hydroxy-Leukotriene E4 | - | 0.00310 | 0.1126 | 1.13 | 1.03 | up | Arachidonic acid metabolism |
| Cucurbitacin A | 葫芦素A | 0.00061 | 0.0591 | 1.18 | 1.02 | up | - |
| Cytidine 3'-Phosphate | 胞苷3 ' -磷酸 | 0.00078 | 0.0665 | 1.14 | 1.02 | up | Pyrimidine metabolism; Metabolic pathways |
| Uridine Monophosphate (Ump) | - | 0.00136 | 0.0833 | 1.17 | 1.02 | up | - |
| Cytosine Arabinoside Monophosphate | - | 0.02268 | 0.2503 | 1.07 | 1.02 | up | - |
| Pyridoxal | 吡哆醛 | 0.00200 | 0.0909 | 1.10 | 1.02 | up | Metabolic pathways; Vitamin B6 metabolism; Microbial metabolism in diverse environments; Biosynthesis of cofactors |
| Forsmann Antigen | - | 0.02411 | 0.2561 | 1.02 | 1.02 | up | - |
| Sedoheptulose | - | 0.04635 | 0.3124 | 1.03 | 1.02 | up | Metabolic pathways;Carbon fixation in photosynthetic organisms |
| Folcepri | - | 0.04587 | 0.3117 | 1.00 | 1.02 | up | - |
| Guanosine 2'-Monophosphate | - | 0.00969 | 0.1801 | 1.09 | 1.02 | up | - |
| 4-Hydroxypyridine | 4-羟基吡啶 | 0.00205 | 0.0909 | 1.10 | 1.02 | up | - |
| Dide-O-Methyl-4-O-Alpha-D-Glucopyranosylsimmondsin | - | 0.02271 | 0.2503 | 1.07 | 1.02 | up | - |
| Cytidine-3'-Monophosphate | - | 0.00879 | 0.1717 | 1.06 | 1.02 | up | Pyrimidine metabolism;Metabolic pathways |
| Cinncassiol A | 辛卡西尔A | 0.03738 | 0.2941 | 1.03 | 1.02 | up | - |
| Protoanemonin | 原白头翁素 | 0.00097 | 0.0719 | 1.12 | 1.02 | up | Chlorocyclohexane and chlorobenzene degradation; Microbial metabolism in diverse environments |
| 4-Hydroxy-6-Methyl-2-Pyrone | 4－羟基－6－甲基－2－吡喃酮 | 0.00212 | 0.0925 | 1.06 | 1.02 | up | - |
| Homatropine | 后马托品 | 0.03029 | 0.2720 | 1.01 | 1.02 | up | - |
| 2-Aminobicyclo[3.1.0]Hexane-2,6-Dicarboxylic Acid | - | 0.00160 | 0.0864 | 1.05 | 1.02 | up | - |
| Uridine 5'-Monophosphate | - | 0.00080 | 0.0665 | 1.04 | 1.02 | up | Pyrimidine metabolism; Metabolic pathways; Nucleotide metabolism; Biosynthesis of cofactors |
| D-Ribulose-1,5-Bisphosphate | - | 0.02119 | 0.2450 | 1.04 | 0.98 | down | Glyoxylate and dicarboxylate metabolism; Biosynthesis of secondary metabolites;Microbial metabolism in diverse environments;Carbon fixation in photosynthetic organisms;Pentose phosphate pathway |
| N-Acetyl-L-Phenylalanine | - | 0.01323 | 0.2079 | 1.08 | 0.97 | down | Metabolic pathways;  Phenylalanine metabolism |
| N-Acetyl-Dl-Valine | - | 0.01446 | 0.2135 | 1.08 | 0.97 | down | - |
| G-Glu-Val | G-谷氨酰-缬氨酸 | 0.04257 | 0.3053 | 1.06 | 0.97 | down | - |
| 5-(3-Pyridyl)-2-Hydroxytetrahydrofuran | - | 0.03639 | 0.2930 | 1.21 | 0.97 | down | - |
| Indoline | 二氢吲哚 | 0.04292 | 0.3053 | 1.17 | 0.97 | down | - |
| O-Acetylserine | O -乙酰丝氨酸 | 0.02239 | 0.2502 | 1.13 | 0.97 | down | Biosynthesis of secondary metabolites;Sulfur metabolism;Microbial metabolism in diverse environments;Cysteine and methionine metabolism; Carbon metabolism; Biosynthesis of various antibiotics;Biosynthesis of amino acids;Sulfur relay system |
| 2,6-Pyridinedicarboxylic Acid | 2,6-吡啶二甲酸 | 0.01758 | 0.2288 | 1.23 | 0.97 | down | - |
| Hypoxanthine | 次黄嘌呤 | 0.02173 | 0.2473 | 1.28 | 0.97 | down | Metabolic pathways; Nucleotide metabolism; Purine metabolism |
| N-Oleoyl Phenylalanine | N -油酰基苯丙氨酸 | 0.03962 | 0.2981 | 1.37 | 0.96 | down | - |
| N-Oleoyl Leucine | N -油酰亮氨酸 | 0.03179 | 0.2779 | 1.34 | 0.96 | down | - |
| 2-[2-(4-Hydroxy-3-Methoxyphenyl)Ethyl]-5-Octylfuran | - | 0.03894 | 0.2963 | 1.41 | 0.96 | down | - |
| Urate Radical | - | 0.01373 | 0.2082 | 1.33 | 0.96 | down | - |
| N-Palmitoyl Phenylalanine | - | 0.03280 | 0.2796 | 1.39 | 0.96 | down | - |
| 2-Hydroxycinnamic Acid | 2 -羟基肉桂酸 | 0.00187 | 0.0892 | 1.48 | 0.96 | down | Metabolic pathways; Phenylalanine metabolism; Biosynthesis of secondary metabolites;Biosynthesis of various plant secondary metabolites;Microbial metabolism in diverse environments |
| Pa(15:0/22:6(5Z,7Z,10Z,13Z,16Z,19Z)-Oh | - | 0.04013 | 0.2987 | 1.40 | 0.96 | down | - |
| 6,8-Dihydroxypurine | 6，8 -二羟基嘌呤 | 0.00764 | 0.1656 | 1.50 | 0.96 | down | - |
| Gamma-Glutamyltyrosine | γ -谷氨酰酪氨酸 | 0.03610 | 0.2919 | 1.40 | 0.96 | down | - |
| L-Aspartic Acid | L-天冬氨酸 | 0.00230 | 0.0966 | 1.58 | 0.96 | down | Biosynthesis of various other secondary metabolites; Biosynthesis of cofactors; Cysteine and methionine metabolism;Carbon metabolism;Biosynthesis of various antibiotics; Biosynthesis of various plant secondary metabolites; Alanine, aspartate and glutamate metabolism; beta-Alanine metabolism; Bacterial chemotaxis; Pantothenate and CoA biosynthesis;ABC transporters;Carbon fixation in photosynthetic organisms; D-Amino acid metabolism; Arginine biosynthesis; Lysine biosynthesis; 2-Oxocarboxylic acid metabolism; Histidine metabolism; Biosynthesis of amino acids;Glycine, serine and threonine metabolism; Monobactam biosynthesis; Biosynthesis of secondary metabolites;Nicotinate and nicotinamide metabolism; Cyanoamino acid metabolism;Two-component system;Aminoacyl-tRNA biosynthesis |
| L-Tyrosine | L-酪氨酸 | 0.00084 | 0.0679 | 1.68 | 0.96 | down | Biosynthesis of secondary metabolites;Tyrosine metabolism;Biosynthesis of cofactors;Thiamine metabolism; Ubiquinone and other terpenoid-quinone biosynthesis;2-Oxocarboxylic acid metabolism; Biosynthesis of various antibiotics;Biosynthesis of various plant secondary metabolites;Biosynthesis of amino acids;Methane metabolism;Cyanoamino acid metabolism; Biosynthesis of various other secondary metabolites; Monobactam biosynthesis; Phenylalanine metabolism; Aminoacyl-tRNA biosynthesis;Biosynthesis of vancomycin group antibiotics;Phenylalanine, tyrosine and tryptophan biosynthesis;Novobiocin biosynthesis;Biosynthesis of enediyne antibiotics |
| Kiwiionoside | b-D-吡喃葡萄糖苷，（1S，3R，4R）-3,4-二羟基-4-[（1E，3R）-3-羟基-1-丁烯-1-基]-3,5,5-三甲基环己基 | 0.02876 | 0.2663 | 1.29 | 0.96 | down | - |
| Oxypurinol | 羟嘌呤醇 | 0.00227 | 0.0966 | 1.57 | 0.96 | down | - |
| Dl-M-Tyrosine | - | 0.00033 | 0.0433 | 1.62 | 0.96 | down | - |
| L-Phenylalanine | L-苯丙氨酸 | 0.00951 | 0.1785 | 1.49 | 0.96 | down | Metabolic pathways; Biosynthesis of various alkaloids;Biosynthesis of various other secondary metabolites;2-Oxocarboxylic acid metabolism;ABC transporters;Biosynthesis of various plant secondary metabolites;Biosynthesis of amino acids;Cyanoamino acid metabolism; Biosynthesis of secondary metabolites;D-Amino acid metabolism;Aminoacyl-tRNA biosynthesis; Phenylalanine, tyrosine and tryptophan biosynthesis; Phenylalanine metabolism |
| Quinolinic Acid | 喹啉酸 | 0.01368 | 0.2082 | 1.41 | 0.96 | down | Metabolic pathways; Nicotinate and nicotinamide metabolism;beta-Alanine metabolism;Tryptophan metabolism;Biosynthesis of cofactors |
| 2,6-Dihydroxypurine | 2，6 -二羟基嘌呤 | 0.00609 | 0.1585 | 1.69 | 0.96 | down | Metabolic pathways; Biosynthesis of secondary metabolites;Purine metabolism;Microbial metabolism in diverse environments;Caffeine metabolism;Nucleotide metabolism |
| 2-(Acetylamino)Hexanoic Acid | - | 0.00944 | 0.1783 | 1.32 | 0.95 | down | - |
| Glutamylvaline | 谷氨酰胺 | 0.01674 | 0.2267 | 1.39 | 0.95 | down | - |
| 8-Hydroxy-7-Methylguanine | - | 0.03622 | 0.2920 | 1.39 | 0.95 | down | - |
| Albaconazole | 阿巴康唑 | 0.01594 | 0.2228 | 1.64 | 0.95 | down | - |
| N-Acetyl-L-Tyrosine | N-乙酰-L-酪氨酸 | 0.00100 | 0.0719 | 1.58 | 0.95 | down | - |
| 2-Phenylacetamide | 2 -苯基乙酰胺 | 0.00145 | 0.0844 | 1.71 | 0.95 | down | Metabolic pathways; Phenylalanine metabolism; Microbial metabolism in diverse environments; Styrene degradation |
| Glutamyltyrosine | 谷氨酰酪氨酸 | 0.02654 | 0.2585 | 1.57 | 0.95 | down | - |
| Uric Acid | 尿酸 | 0.00095 | 0.0719 | 1.98 | 0.95 | down | Metabolic pathways;Purine metabolism;Microbial metabolism in diverse environments |
| O-Desmethyl Gefitinib | O -去甲基吉非替尼 | 0.01890 | 0.2349 | 1.49 | 0.95 | down | - |
| 4'-O-Methyldelphinidin 3-O-Beta-D-Glucoside | - | 0.00164 | 0.0865 | 1.97 | 0.94 | down | - |
| Nadp+ | - | 0.03935 | 0.2971 | 1.68 | 0.94 | down | Metabolic pathways; Glutathione metabolism; Drug metabolism - other enzymes;Biosynthesis of cofactors;Nicotinate and nicotinamide metabolism; Photosynthesis |
| Beta-Alanyl-L-Arginine | - | 0.01360 | 0.2079 | 1.68 | 0.94 | down | Metabolic pathways; beta-Alanine metabolism |
| Timonacic | 噻莫西酸 | 0.03147 | 0.2761 | 1.73 | 0.94 | down | - |
| Quisqualic Acid | 使君子氨酸 | 0.03580 | 0.2909 | 1.52 | 0.94 | down | - |
| Adp-Ribose 1'-2' Cyclic Phosphate | - | 0.04957 | 0.3197 | 1.67 | 0.94 | down | - |
| 3-[(2-Oxoacetyl)Oxy]-4-(Trimethylazaniumyl)Butanoate-Coa | - | 0.02134 | 0.2456 | 1.94 | 0.93 | down | - |
| Leucodopachrome | 白细胞色素 | 0.04439 | 0.3074 | 1.40 | 0.93 | down | Metabolic pathways; Biosynthesis of secondary metabolites;Tyrosine metabolism |
| Pyochelin | 2-甲基-1H-咪唑-4-甲酸 | 0.00861 | 0.1698 | 1.82 | 0.93 | down | Biosynthesis of siderophore group nonribosomal peptides;Biosynthesis of secondary metabolites |
| Cefroxadine | - | 0.01085 | 0.1908 | 1.84 | 0.93 | down | - |
| Pe(18:1(9Z)/16:0) | - | 0.03681 | 0.2937 | 1.85 | 0.92 | down | Metabolic pathways; Glycerophospholipid metabolism;Biosynthesis of secondary metabolites; Pathogenic Escherichia coli infection |
| Simmondsin | 西蒙得木素 | 0.00599 | 0.1584 | 2.02 | 0.92 | down | - |
| Nadp | - | 0.04293 | 0.3053 | 1.92 | 0.92 | down | Metabolic pathways; Glutathione metabolism; Drug metabolism - other enzymes;Biosynthesis of cofactors;Nicotinate and nicotinamide metabolism; Photosynthesis |
| Dihydrozeatin | 二羟玉米素 | 0.01831 | 0.2314 | 2.07 | 0.91 | down | Biosynthesis of secondary metabolites |
| N-(2,6-Difluorophenyl)-2-Oxoglycyl-N-((1S)-1-(Carboxymethyl)-2-Oxo-3-(2,3,5,6-Tetrafluorophenoxy)Propyl)-L-Alaninamide | - | 0.01036 | 0.1866 | 2.10 | 0.91 | down | - |
| Pantothenic Acid | 泛酸 | 0.03310 | 0.2803 | 1.89 | 0.90 | down | Metabolic pathways; Biosynthesis of secondary metabolites;Pantothenate and CoA biosynthesis; beta-Alanine metabolism; Biosynthesis of cofactors |
| Alpha-Cyano-4-Hydroxycinnamic Acid | - | 0.01634 | 0.2252 | 1.90 | 0.90 | down | - |
| N-Acetyl-L-Methionine | - | 0.00666 | 0.1604 | 2.15 | 0.90 | down | - |
| Fluazifop | 吡氟禾草灵 | 0.01780 | 0.2300 | 2.22 | 0.90 | down | - |
| Sucralose | 三氯蔗糖 | 0.00690 | 0.1608 | 2.11 | 0.89 | down | - |
| Isopentenyl Adenosine | - | 0.01539 | 0.2213 | 2.15 | 0.89 | down | Biosynthesis of secondary metabolites |
| 13-Oxoode | 13-氧代 | 0.02164 | 0.2472 | 2.12 | 0.88 | down | Linoleic acid metabolism |
| 3H-1,2,4-Triazol-3-One, 4-(4-(4-((1R,2R)-2-(2,4-Difluorophenyl)-2-Hydroxy-1-Methyl-3-(1H-1,2,4-Triazol-1-Yl)Propyl)-1-Piperazinyl)Phenyl)-2,4-Dihydro-2-((4-(Trifluoromethoxy)Phenyl)Methyl)- | - | 0.00018 | 0.0340 | 2.86 | 0.87 | down | - |
| 3,5-Dihydroxydecanoic Acid | 3，5 -二羟基癸酸 | 0.00264 | 0.1032 | 2.21 | 0.87 | down | - |
| Wistin | - | 0.01687 | 0.2278 | 2.42 | 0.87 | down | - |
| Orotic Acid | 乳清酸 | 0.02613 | 0.2585 | 2.43 | 0.86 | down | Pyrimidine metabolism;Metabolic pathways;Biosynthesis of cofactors |
| Cmp-2-Aminoethylphosphonate | - | 0.01142 | 0.1950 | 2.50 | 0.85 | down | Metabolic pathways;Phosphonate and phosphinate metabolism |
| Benzyladenine 7-O-Beta-D-Glucoside | - | 0.00029 | 0.0400 | 2.90 | 0.85 | down | - |
| Vomifoliol 9-[Glucosyl-(1->4)-Xylosyl-(1->6)-Glucoside] | - | 8.00E-05 | 0.0275 | 3.34 | 0.82 | down | - |
| 4-Hydroxytriazolam | - | 0.00046 | 0.0515 | 3.51 | 0.75 | down | - |

**Table S3. Plasmids and strains used in this study.**

| **Strains or plasmids** | **Relevant genotype or characteristic** | **Source** |
| --- | --- | --- |
| **Plasmids** | | |
| pAK1900 | *E. coli–P. aeruginosa* shuttle cloning vector carrying P*lac* upstream of MCS; Cb^r^ | Lab stock |
| pET28a | T7 *lac* promoter-operator, N-terminal His tag; kn^r^ | Lab stock |
| pEX18Ap | Gene replacement vector from pUC18; sacB^+^Apr | Lab stock |
| pMS402 | *lux-*based promoter reporter plasmid, Kn^r^ | Lab stock |
| mini-CTX-lacZ | Integration plasmid; Tc^r^ | Lab stock |
| mini-CTX-lacZ-*hutG*  -flag | mini-CTX-lacZ containing *hutG* promoter and the entire *hutG* gene fused with FLAG tag at C-terminal; Tc^r^ | This study |
| pEX18Ap::*hutC* | pEX18Ap derivative, for replacing PAO1 *hutC* gene; cb^r^ | This study |
| pEX18Ap::*hutH* | pEX18Ap derivative, for replacing PAO1 *hutH* gene; cb^r^ | This study |
| pEX18Ap::*hutI* | pEX18Ap derivative, for replacing PAO1 *hutI* gene; cb^r^ | This study |
| pEX18Ap::*hutG* | pEX18Ap derivative, for replacing PAO1 *hutG* gene; cb^r^ | This study |
| pEX18Ap::*hutU* | pEX18Ap derivative, for replacing PAO1 *hutU* gene; cb^r^ | This study |
| pEX18Ap::*hutF* | pEX18Ap derivative, for replacing PAO1 *hutF* gene; cb^r^ | This study |
| p*-hutC* | pAK1900::*hutC*; pAK1900 derivative carrying *hutC* gene on a *ca*. 853 bp *Hin*dIII/*Bam*HI fragment in same orientation as p*lac* | This study |
| p-*hutH* | pAK1900::*hutH*; pAK1900 derivative carrying *hutH* gene on a *ca*. 1.63 kb *Hin*dIII/*Xbal*I fragment in same orientation as p*lac* | This study |
| p-*hutI* | pAK1900::*hutI*; pAK1900 derivative carrying *hutI* gene on a *ca*. 1.33 kb *Hin*dIII/*Bam*HI fragment in same orientation as p*lac* | This study |
| p-*hutG* | pAK1900::*hutG*; pAK1900 derivative carrying *hutG* genes on a *ca*. 9.18 bp *Hin*dIII/*Bam*HI fragment in same orientation as p*lac* | This study |
| p-*hutU* | pAK1900::*hutU*; pAK1900 derivative carrying *hutU* gene on a *ca*. 2.01 kb *Hin*dIII/*Xbal*I fragment in same orientation as p*lac* | This study |
| p-*hutF* | pAK1900::*hutF*; pAK1900 derivative carrying *hutF* gene on a *ca*. 1.46 kb *Hin*dIII/*Bam*HI fragment in same orientation as p*lac* | This study |
| pMS402::*hutH-lux* | pMS402 containing *hutH* promoter region (from -95 to +1 of the start codon) | This study |
| pMS402::*hutG-lux* | pMS402 containing *hutG* promoter region (from -116 to +1 of the start codon) | This study |
| pMS402::*hutU-lux* | pMS402 containing *hutU* promoter region (from -324 to +1 of the start codon) | This study |
| pMS402::*hutF-lux* | pMS402 containing mutated *hutF* promoter region (from -99 to +1 of the start codon, | This study |
| pET28a::His_6_-*hutC* | pET28a derivative carrying *hutC* | This study |
| ***P. aeruginosa* strains** | | |
| PAO1 | Wild type | Lab stock |
| Δ*hutC* | *hutC* deletion mutant of PAO1 | This study |
| Δ*hutC*/p-*hutC* | Δ*hutC* mutant carrying p-*hutC* plasmid | This study |
| Δ*hutH* | *HutH* deletion mutant of PAO1 | This study |
| Δ*hutH*/p-*hutH* | Δ*hutH* mutant carrying p-*hutH* plasmid | This study |
| Δ*hutI* | *HutI* deletion mutant of PAO1 | This study |
| Δ*hutI/*p-*hutI* | Δ*hutI* mutant carrying p-*hutI* plasmid | This study |
| Δ*hutG* | *hutG* deletion mutant of PAO1 | This study |
| Δ*hutG*/p-*hutG* | *hutG* mutant carrying p-*hutG* plasmid | This study |
| Δ*hutU* | *hutU* deletion mutant of PAO1 | This study |
| Δ*hutU*/p-*hutU* | Δ*hutU* mutant carrying p-*hutU* plasmid | This study |
| Δ*hutF* | *hutF* deletion mutant of PAO1 | This study |
| Δ*hutF*/p-*hutF* | Δ*hutF* mutant carrying p-*hutF* plasmid | This study |
| ***E. coli*** | | |
| DH5ɑ | *F– φ80lacZ ΔM15 Δ(lacZYA-argF)U169 recA1 endA1 hsdR17(rk– mk+)phoA supE44 thi-1 gyrA96 relA1 tonA* | Stratagene |
| BL21 (DE3) | *F^–^ ompT hsdSB* (rB^-^ mB^-^) *gal dcm* *met*(DE3) | Invitrogen |

**Table S4. Primers used in this study**

| **Primer** | **Sequence (5' to 3')** | **Application** |
| --- | --- | --- |
| pEX-*hutC*-up-F | TTAGGATCCTAAGCAAGCGCAACGCG | Constructing *hutC* |
| pEX-*hutC*-up-R | TAATCTAGATCGCCGAGTTGCGAGGCC | deletion mutant |
| pEX-*hutC*-down-F | ATATCTAGATGCTGCACCCGGGTTCCC |  |
| pEX-*hutC*-down-R | AATAAGCTTACCGATGGGGTCGGGGTT |  |
| *hutC*-test-F | TCGCGCCAGGTCCAGAAACT | Test *hutC* mutant |
| *hutC*-test-R | GCATAGATCAGGTTGAAGTCGCG |  |
| pEX-*hutH*-up-F | TTAGGATCCTCGACTACATCCCGGCG | Constructing *hutH* |
| pEX-*hutH*-up-R | TAATCTAGAAGGTCGGCCAGGGTCAACTG | deletion mutant |
| pEX-*hutH*-down-F | ATATCTAGATTGCCTGAACGCGCTGCTG |  |
| pEX-*hutH*-down-R | AATAAGCTTTATTGGCCGGCGGCTC |  |
| *hutH*-test-F | GAATCGCCGTCTATACCCTCG | Test *hutH* mutant |
| *hutH*-test-R | GTAATGGCCGAACGAGCC |  |
| pEX-*hutI*-up-F | TTAGGATCCTCCGCTGCGTTGGC | Constructing *hutI* |
| pEX-*hutI*-up-R | TAATCTAGATGCCGCCCTTGAGGGTGG | deletion mutant |
| pEX-*hutI*-down-F | ATATCTAGAACGCGTCATTCGCCACGC |  |
| pEX-*hutI*-down-R | AATAAGCTTTCGCGGGCCGGGTAC |  |
| *hutI*-test-F | GGTGGTCGACAACTTCGTCACC | Test *hutI* mutant |
| *hutI*-test-R | TGCTGTACAGCGGCTTGTCG |  |
| pEX-*hutG*-up-F | TTAGGATCCTCCAGGAGGACCACCTGAG | Constructing *hutG* |
| pEX-*hutG*-up-R | TAATCTAGATCAGTAGCGGCACGCGGC | deletion mutant |
| pEX-*hutG*-down-F | ATATCTAGACTGCTGGAAAGCCTCCTCGCC |  |
| pEX-*hutG*-down-R | AATAAGCTTCTGGCCGAGGCGATCCC |  |
| *hutG*-test-F | CTTCTACCTGTTGCGCGAGACC | Test *hutG* mutant |
| *hutG*-test-R | CGGAAGGCGAGTACCTGGAAGT |  |
| pEX-*hutU*-up-F | TTAGGTACCATCCCGCCCTGGCTG | Constructing *hutU* |
| pEX-*hutU*-up-R | TAATCTAGAGGCGCGGATTTCAATATCGC | deletion mutant |
| pEX-*hutU*-down-F | ATATCTAGAGCGAAGGAGCAGGGCCTGA |  |
| pEX-*hutU*-down-R | AATAAGCTTTCATTCGCGCCGGGG |  |
| *hutU*-test-F | TTACCCGCAATTGCGCTGA | Test *hutU* mutant |
| *hutU*-test-R | AGCGACCAGAACACGTCGC |  |
| pEX-*hutF*-up-F | TTAGGATCCGGATAGACAAAAGCGC | Constructing *hutF* |
| pEX-*hutF*-up-R | TAATCTAGAAGCCTTCCGGCAGCAA | deletion mutant |
| pEX-*hutF*-down-F | ATATCTAGACATTCGTCCAGGTGCTGG |  |
| pEX-*hutF*-down-R | AATAAGCTTTTGCAGGAGTGGACG |  |
| *hutF*-test-F | GATTGATGGTCATGCGACTGAAGCC | Test *hutF* mutant |
| *hutF*-test-R | TGGTGGGCAATCCGAACCGTGA |  |
| *hutC-*comp-F | AATAAGCTTAATCAGCTCACACGCAGTCTG | Constructing *hutC* |
| *hutC*-comp-R | ATTGGATCCTCATGAGCTGAAACGTCC | complementation plasmid |
| *hutH-*comp-F | AATAAGCTTGACGAATTTCCAGGGCG | Constructing *hutH* |
| *hutH*-comp-R | ATTTCTAGATCAAAGACTCGGCAACAGCC | complementation plasmid |
| *hutI-*comp-F | AATAAGCTTCGGAACCGACGGCGG | Constructing *hutI* |
| PA5096-overlap-*hutI*-R | GGTTCGACGCTCCAGATGCACG | complementation plasmid |
| PA5096-overlap-*hutI*-F | TGCATCTGGAGCGTCGAACCATGAAACGACTCTGG |  |
| *hutI*-comp-R | ATTGGATCCTCATCCACGGTATACCTCCTCG |  |
| *hutG-*comp-F | AATAAGCTTCGGAACCGACGGCGG | Constructing *hutG* |
| PA5096-overlap-*hutG*-R | GGTTCGACGCTCCAGATGCACG | complementation plasmid |
| PA5096overlap-*hutG*-F | TGCATCTGGAGCGTCGAACCGTGGATGAAGTCCTGA |  |
| *hutG*-comp-R | ATTGGATCCTCAGGCGTAGCGCTCG |  |
| *hutU-*comp-F | AATAAGCTTACGGTGTTACCGAAGGCCC | Constructing *hutU* |
| *hutU*-comp-R | ATTTTCTAGACAGCGCTGGGCGGTG | complementation plasmid |
| *hutF-*comp-F | AATAAGCTTGGAGAGACTCCTTGTCGAGG | Constructing *hutF* |
| *hutF*-comp-R | ATTGGATCCTCAGTCGAGCAGTTCCCC | complementation plasmid |
| mini-*hutG*-flag-F | AATCTCAGACGGAACCGACGGCGG | Western-blot |
| mini-*hutG*-flag-overlap-R | GGTTCGACGCTCCAGATGCACG |  |
| mini-*hutG*-flag-overlap-F | TGCATCTGGAGCGTCGAACCGTGGATGAAGTCCTGA |  |
| mini-*hutG*-flag-R | ATTAAGCTTGGCGTAGCGCTCGCGACC |  |
| *hutH*-*lux*-F | AATCTCAGAAGACGAATTTCCAGGGCG | Constructing *hutH* |
| *hutH*-*lux*-R | ATTGGATCCGGTTTCAGATGCAGGCTC | promoter plasmid |
| *hutG*-*lux*-F | AATCTCAGATGCTTCAGGCCCAGGG | Constructing *hutG* |
| *hutG*-*lux*-R | ATTGGATCCATCGACTTCTTGTATCGCATG | promoter plasmid |
| *hutU*-*lux*-F | AATCTCAGAGTTACCCGCAATTGCGC | Constructing *hutU* |
| *hutU*-*lux*-R | ATTGGATCCAATTTGCTCGGGGTGG | promoter plasmid |
| *hutF-lux*-F | AATCTCAGAAATCAGCTCACACGCAGTC | Constructing *hutF* |
| *hutF*-*lux*-R | ATTGGATCCGGAGAGACTCCTTGTCG | promoter plasmid |
| *q-hutH*-F | AGCGTTCCATCGTCCTGTC | qRT-PCR |
| *q-hutH*-R | GCACCCACCGAGCCTTT |  |
| *q-fleS*-F | TGCTCTGGCGTGAGGTGA |  |
| *q-fleS*--R | GGGTATCGGTGAGGTCGTT |  |
| *q-flgB-*F | CACCAACGAGCGGCATAT |  |
| *q-flgB*-R | TGAAGGACGCCTGGAAGT |  |
| *q-minC*-F | ACCGCTGGACATCAAGGA |  |
| *q-minC*-R | GAGGTCGGCTTGGCTCAT |  |
| *q-hutG*-F | GCTACCTGGCCGAAGTGT |  |
| *q-hutG*-R | TTGGTGCCGAGATTGAAGT |  |
| *q-pilA*-F | CCGCTGAAGACCACTG |  |
| *q-pilA*-R | ATCACCCGCACCACTA |  |
| pET28a-*hutC*-F | AATGGATCCGTGACGTCCTCTTCCTC | Protein cloning |
| pET28a-*hutC*-R | ATTAAGCTTTCATGAGCTGAAACGTCCTTC |  |
| EMSA*-hutH*-F | GGTACTGGCTGTCCGGGTCG | EMSA |
| EMSA*-hutH*-R | AGACCAGCGATGCATTAGACGATT |  |
| EMSA-*hutG*-F | GTAGGTGTCCTCTTCGGCA |  |
| EMSA-*hutG*-R | GACAGAACGTTCCCTCTTC |  |
| EMSA-*hutU*-F | CCCTTATTCCTTTTATTGGGTG |  |
| EMSA-*hutU*-R | AGTCGCTACACCTGAAGGCGC |  |
| EMSA-*hutF*-F | CCGTTGCTCCTTAGCAGCG |  |
| EMSA-*hutF*-R | CGGTTACCTCTTGCACGCG |  |
| *hutG*_-108 to -1_-F | ACGGCGGCCTGAGCC | EMSA |
| *hutG*_-108 to -1_-R | GGTTCGACGCTCCAGATG |  |
| *hutG*_-90 to -1_-F | GCCAATAACAATGATCGAGGCCG |  |
